# Peri‐Microvascular Glycogen and Lactate Regulate Capillary Constrictions and Ischemia Outcome in Mice

**DOI:** 10.1111/jnc.70430

**Published:** 2026-04-16

**Authors:** Gokhan Uruk, Buket Donmez‐Demir, Sinem Yilmaz‐Ozcan, Canan Cakir‐Aktas, Aslihan Taskiran‐Sag, Gokce Gurler, Jordi Duran, Joan J. Guinovart, Otto Baba, Tsuyoshi Morita, Hulya Karatas, Turgay Dalkara, Muge Yemisci

**Affiliations:** ^1^ Institute of Neurological Sciences and Psychiatry Hacettepe University Ankara Turkey; ^2^ Institut Químic de Sarrià (IQS) Universitat Ramon Llull (URL) Barcelona Spain; ^3^ Institute for Research in Biomedicine (IRB Barcelona) Barcelona Spain; ^4^ Oral and Maxillofacial Anatomy Tokushima University Graduate School Tokushima Japan; ^5^ Department of Neurology, Faculty of Medicine Hacettepe University Ankara Turkey

**Keywords:** CD13‐positive pericyte, glycogen, ischemic stroke, L‐lactate, microcirculation

## Abstract

Ischemic stroke results in sudden blood flow cessation, thus unmet energy requirements. Glycogen stored around peri‐microvascular astrocyte end‐feet may mediate capillary contractility and cerebral blood flow alterations. Under glucose‐deprived and hypoxic conditions, lactate derived from these glycogen stores may serve as an emergency fuel to sustain tissue perfusion during an acute period of ischemic stroke. To elucidate the impact of glycogen utilization on brain microcirculation, both 1,4‐dideoxy‐1,4‐imino‐d‐arabinitol hydrochloride (DAB) administered to wild‐type (WT) intracerebroventricularly (i.c.v.), and central nervous system and astrocyte‐specific glycogen synthase‐1 knock‐out (GYS1^Nestin‐KO^ and GYS1^Gfap‐KO^) mice were used. We assessed regional cerebral blood flow changes in vivo, pericyte‐associated microvascular constrictions, semi‐quantitative peri‐microvascular glycogen levels, and lactate transporters ex vivo. Experiments revealed that both pharmacological and genetic manipulations of glycogen metabolism also resulted in severely compromised blood flow dynamics and higher infarct volumes after stroke. Disrupted cerebral glycogen utilization induced CD13‐positive pericyte‐associated microvascular constrictions, which were highly correlated with peri‐microvascular periodic acid Schiff (PAS), IV58B6, and ESG1A9 intensity levels. Lastly, intravenous (i.v.) D/L‐lactate and i.c.v. L‐lactate administration reversed microvascular constrictions while glycogen phosphorylase inhibition potently reduced microvascular monocarboxylate transporter‐1 (MCT1) coverage. In conclusion, disrupted glycogen utilization causes ischemic‐like microvascular constrictions, increases susceptibility to brain ischemia, and is reversible with systemic lactate administration. Understanding the role of glycogen and lactate metabolism at the neurogliovascular level in the brain may provide novel insight into the pathophysiology and therapeutic opportunities of cerebrovascular disorders.

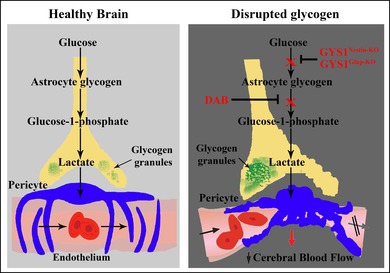

## Graphical abstract

Disruption of astrocytic glycogen metabolism, induced by glycogen phosphorylase inhibition (DAB) or genetic deficiency of GYS1 (GYS1^Nestin‐KO^ and GYS1^Gfap‐KO^), reduces lactate availability to pericytes and impairs microvascular function. This metabolic deficit leads to pericyte‐associated capillary constriction, reduced cerebral blood flow, and increased susceptibility to ischemic injury. Notably, both intracerebroventricular L‐lactate and intravenous L‐ and D‐lactate administration reverse these constrictions, highlighting a critical role for glycogen‐derived lactate in maintaining capillary patency and microvascular stability.

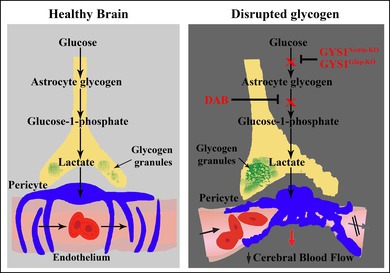

**Abbreviations**
ANLS: Astrocyte neuron lactate shuttle
CCA: Common carotid artery
CCD: Charge‐coupled device
CD13: Cluster of Differentiation‐13
CNS: Central Nervous System
DAB: 1,4‐dideoksi‐1,4‐imino‐d‐arabinitol hydrochloride
Dimedone: 5,5‐Dimethyl‐1,3‐cyclohexanedione
ECA: External Carotid Artery
FeCl_3_
: Ferric Chloride
GFAP: Glial Fibrillary Acidic Protein
GP: Glycogen phosphorylase
GPR81: G protein‐coupled receptor 81
GYS‐1: Glycogen synthase‐1
GYS1^Gfap‐KO^
: Glycogen synthase‐1 GFAP Knock‐out
GYS1^Nestin‐KO^
: Glycogen synthase‐1 Nestin Knock‐out
HK2: Hexokinase‐2
i.c.v: intracerebroventricular
i.v: intravenous
I/R: Ischemia/Recanalization
ICT: Inverse Correlation Time
LSCI: Laser Speckle Contrast Imaging
LSF: Laser‐Speckle Flowmetry
MCA: Middle Cerebral Artery
MCAo: Middle Cerebral Artery occlusion
MCT‐1: Monocarboxylate transporter‐1
MCT‐12: Monocarboxylate transporter‐12
MCT‐4: Monocarboxylate transporter‐4
NG2: Neural‐glial antigen‐2
NIBP: Non‐Invasive Blood Pressure
NO: Nitric Oxide
PAS: Periodic Acid Schiff
PDGFR‐β: Platelet‐derived growth factor receptor‐beta
rCBF: Regional Cerebral Blood Flow
RRID: Research Resource Identifier

## 1 Introduction

The human brain comprises 2% of the body's weight; however, it accounts for 20%–25% of its resting oxygen metabolism and glucose consumption (Attwell and Laughlin 2001; Magistretti and Allaman 2015). Neurons exclusively rely on constant glucose flux and harbor a negligible amount of glycogen as a readily usable glucose store. However, glial cells contain glycogen, and although the amount is detected less compared to other tissues, this energy reserve could support the metabolism for a short period of time when needed (Dringen et al. 1993; Pellerin et al. 2007; Pellerin and Magistretti 1994). Ischemic stroke results in an abrupt oxygen and glucose insufficiency to the glial cells and neurons; thus, drastic metabolic changes occur (Bouma et al. 1992; De Keyser et al. 2008; Lipton 1999; Magistretti et al. 1999; Pellerin and Magistretti 1994). During ischemia, neurons cannot obtain glucose from blood, and the glycogen stored in the cells cannot meet the energy requirements for more than a few seconds (Saez et al. 2014). Therefore, glial cells, especially astrocytes, play a key role in supporting neurons also under ischemic conditions via the astrocyte‐neuron lactate shuttle (Fern 2015; Gurer et al. 2009; Obel et al. 2012). Glial glycogen reserve can metabolically support the tissue by switching glycolysis from blood glucose to glycogen‐derived glucose (Rothman and Dienel 2019). The steady and dynamic balance between glycogenesis and glycogenolysis provides the endogenous energy reserve in ischemia (Bak et al. 2018).

Patency of microvasculature is essential for reperfusion, hence survival in cerebral ischemia (Hall et al. 2014; Peppiatt et al. 2006; Yemisci et al. 2009). Our previous studies demonstrated that microvascular constrictions due to ischemia are spatially correlated with contracted pericytes in the brain and the retina (Alarcon‐Martinez et al. 2019; Yemisci et al. 2009). Although the pathophysiological mechanisms underlying the control of microvascular functions are not fully understood in the brain yet, pericytes are shown to have a role in mediating microvascular constrictions in the metabolically compromised retina (Alarcon‐Martinez et al. 2019; Attwell and Laughlin 2001; Hall et al. 2014; Kisler et al. 2017; Mishra et al. 2016; Yemisci et al. 2009).

Glycogen metabolism is tightly regulated in each cell (Dringen et al. 1993; Pellerin et al. 1997; Swanson 1992). Glycogen synthase (GYS‐1) is the rate‐limiting enzyme in adding glucose moieties to existing glycogen granules, thus glycogenesis, and Glycogen phosphorylase (GP) is the key enzyme in glycogen breakdown, glycogenolysis (Zois and Harris 2016). In this study, we aimed to investigate the effects of pharmacologically or genetically disrupted peri‐microvascular glycogen and superimposed permanent cerebral ischemia on microvasculature. To disrupt glycogen utilization pharmacologically, we administered 1,4‐dideoxy‐1,4‐imino‐d‐arabinitol hydrochloride (DAB) intracerebroventricularly (i.c.v.), which potently inhibits GP enzyme activity and prevents the use of glycogen (Kilic et al. 2018). For genetic disruption, we used central nervous system‐specific (GYS1^Nestin‐KO^) and astrocyte‐specific (GYS1^Gfap‐KO^) Glycogen Synthase‐1 knockout mice (Duran et al. 2013; Matsui et al. 2017). We used a permanent cerebral ischemia model to mimic the clinics of ischemic stroke patients without recanalization (Hackett et al. 2016). We also employed i.c.v. and intravenous (i.v.) L/D‐lactate administrations along with i.c.v. DAB to demonstrate the reversibility of GP inhibition and its impact on microvascular constrictions. Finally, we demonstrated ischemia/recanalization (I/R) to show whether the microvascular constrictions reverse once glycogen utilization is established.

We found that disruption of glycogen metabolism either by i.c.v. DAB administration to mice or in both GYS1^Gfap‐KO^ and GYS1^Nestin‐KO^ mice induced microvascular constrictions near CD13‐positive pericytes as observed in ischemia. Interestingly, when permanent cerebral ischemia was introduced to mice with already impaired glycogen utilization, CD13‐positive pericyte constrictions and infarct volume were increased compared to wild‐type mice. When either L/D‐lactate i.v. or L‐lactate i.c.v. administered as an energy source to bypass the inhibition of GP (Allaman et al. 2011; Berthet et al. 2009; Kilic et al. 2018) DAB‐induced CD13‐positive pericyte constrictions were diminished. I/R after DAB injections revealed reversible microvascular constrictions even after ischemic insult, coinciding with the reversal of GP inhibition. Microvascular monocarboxylate transporter‐1 (MCT‐1) coverage was diminished with DAB administration, and in GYS1^Nestin‐KO^. Disruption of glycogen utilization causes energy deficiency in the brain, resembles ischemia, and leads to increased microvascular constrictions. As we previously observed in the mouse retina, these microvascular constrictions suggest a role for CD13‐positive capillary pericyte contractions, which spatially correspond to ischemia‐induced peri‐microvascular glycogen depletion (Alarcon‐Martinez et al. 2019). We suggest that insufficient peri‐microvascular glycogen utilization may have a role in the pathophysiology of incomplete reperfusion after cerebral ischemia.

## 2 Materials and Methods

### 2.1 Animals and Study Approval

The experiments were performed in adult (20–30 g) male and female Swiss albino (*n* = 3/group), C57Bl/6J wild‐type (WT) (*n* = 3/group), GYS1^Nestin‐KO^ (*n* = 3/group), and GYS1^Gfap‐KO^ (*n* = 3/group) mice, which were housed under diurnal lighting conditions (12 h light and 12 h darkness) at room temperature (22°C ± 2°C, 50%–60% humidity). The animals had ad libitum access to water and food. Initial breeding pairs of GYS1^Nestin‐KO^ mice (Duran et al. 2013) and fixed and frozen brain tissues of GYS1^Gfap‐KO^ were obtained from Dr. Duran and Dr. Guinovart at IRB Barcelona, and the colony was further expanded, genotyped, and maintained at Hacettepe University Institute of Neurological Sciences and Psychiatry. Animal housing, care, and the experimental procedures were all done in accordance with institutional guidelines. A maximum of 5 mice in a single large Eurostandard Type 2 L cage were kept together for up to 6 months, 3 weeks after weaning. Cages were made of transparent polycarbonate and were autoclaveable. All procedures were approved by Hacettepe University Animal Experimentations Local Ethics Board (Registration number: 2016–60/3 and 2018–56/06). The experiments have been reported in compliance with the ARRIVE Guidelines. Animals were assigned to experimental groups using a predefined, sex‐balanced allocation scheme (fixed numbers of male and female animals per group), rather than a randomization procedure. Group composition was determined a priori to ensure comparable sex distribution across experimental conditions.

For all the surgical procedures mentioned below, mice were anesthetized with isoflurane (4%–5% induction dose, followed by 1.5%–2% maintenance). Corneal reflex and hind‐paw nociceptive reflex sensitivity were assessed periodically for depth of anesthesia. O_2_ flow rate was maintained around 2 L/min via a facemask for the duration of the experiment to maintain tissue O_2_ saturation above 92% to avoid anesthesia‐induced hypoxia. Body temperature was monitored with a rectal probe and maintained at 37.0°C ± 0.2°C by a homeothermic blanket control unit (Harvard Apparatus, USA). Pulse rate and oxygen saturation were monitored with an oximeter (The LifeSense VET Pulse Oximeter, Nonin Medical Inc., USA) from the right lower limb throughout the experiment. Mice were placed on the heating blanket until fully recovered from anesthesia. The animals that developed hemodynamic instability during experimental procedures were excluded from the study. Only a total of 6 mice (4 GYS1^Nestin‐KO^, 2 i.c.v. DAB‐injected) were excluded during ischemia procedures.

### 2.2 Intracerebroventricular (i.c.v.) Injection

Mice were placed in a rodent stereotaxic frame (WPI, USA) in a prone position under isoflurane. The cranium was exposed with a vertical skin incision, and a burr hole was opened over the appropriate hemisphere (right hemisphere for filament MCAo experiments; left hemisphere for intracerebroventricular injections). A 10 μL 26‐gauge Hamilton syringe was placed reaching the ventricle (coordinates respective to Bregma: −0.15 mm posterior, 0.7 mm lateral, 3.15 mm of depth) (Kawasaki et al. 2016) under a surgical stereomicroscope (Nikon SMZ 1000, Nikon). To minimize tissue trauma due to injection, the syringe was slowly advanced to the target depth over 10 min. After waiting for 5 min, either DAB alone (*n* = 3; 0.75 μL of 0.25 M DAB in saline), or a combination of DAB and sodium D/L‐lactate (*n* = 3 per group; total injection volume 0.75 μL, consisting of 0.25 μL of 0.25 M DAB and 0.5 μL of 0.5 M sodium D/L‐lactate in saline) was injected intracerebroventricularly over 5 min at a rate of 0.15 μL/min. This corresponds to an approximate intracerebroventricular dose of 1.4–2.1 mg/kg sodium D/L‐lactate in adult mice weighing 20–30 g. The lactate concentration (0.5 M) was selected based on prior metabolic superfusion studies from our laboratory (Kilic et al. 2018), whereas the intracerebroventricular delivery paradigm was adapted from established ischemia models demonstrating neuroprotective effects of centrally administered lactate (Berthet et al. 2009). Following another 5 min wait after the i.c.v. injections, the syringe was removed within 10 min. The midline incision was sutured via 4–0 nylon suture.

### 2.3 Intravenous (i.v.) Lactate Administration

For intravenous D/L‐lactate experiments, mice received 5 μL per gram of body weight of either sodium D/L‐lactate solutions (200 mmol/L in saline) injected randomly in the tail vein at reperfusion using a 1‐mL syringe with a 25‐gauge needle (Castillo et al. 2015). This corresponds to an approximate systemic dose of ~112 mg/kg sodium D/L‐lactate. Prilocaine was injected subcutaneously to provide pain relief. Mice were placed on the heating blanket until they were fully recovered from anesthesia. All surgeries were performed during daylight, between 9 am and 5 pm. Each mouse was considered an experimental unit.

### 2.4 Thrombotic Middle Cerebral Artery (MCA) Occlusion (MCAo)

Thrombotic Middle cerebral artery occlusion (MCAo) was performed as previously described (Karatas et al. 2011). Mice were placed in a stereotaxic frame (WPI, USA), the scalp was opened, and the cranial sutures and bregma were exposed. The right temporal muscle was bluntly dissected until the squamous part of the temporal bone was exposed. The area just above the junction between the zygomatic arch and the squamous bone was thinned using a high‐speed drill and cooled with saline. The trace of MCA was visualized through the thinned temporal bone. A thin bony film was lifted by forceps to open the burr hole. After obtaining a stable 10‐min epoch of the preischemic regional cerebral blood flow (rCBF) via laser‐speckle flowmetry (LSF), 30% ferric chloride (FeCl_3_)‐saturated filter paper (0.3 × 1 mm) was placed over the MCA. The filter paper was removed after 3–10 min of placement on observing the cessation of rCBF due to successful clot formation (Karatas et al. 2011). MCAo was performed for 2 h, and rCBF was continuously monitored during the occlusion and 115 min after.

### 2.5 Ischemia/Recanalization via Proximal Middle Cerebral Artery Occlusion (pMCAo)

Transient focal cerebral ischemia‐recanalization was performed via proximal middle cerebral artery occlusion (pMCAo) in i.c.v. DAB‐injected mice. Focal ischemia was induced by an intraluminal monofilament technique, as previously described (Yemisci et al. 2009). Mice were placed in a supine position under isoflurane anesthesia. Briefly, a midline neck incision was made, and paratracheal muscles were carefully dissected. The right common, external, and internal carotid arteries were exposed, common and external carotid arteries were meticulously isolated from the vagus nerve and then ligated with 5–0 nylon suture proximally. A small incision was made through the internal carotid artery 1 mm proximal to the carotid bifurcation. 8–0 nylon monofilament (Ethilon; Ethicon, Norderstedt, Germany) coated with silicon resin (Xantopren: Bayer Dental, Osaka, Japan; diameter 170–200 lm) was introduced through a small incision into the internal carotid artery and advanced 10 mm distal to the carotid bifurcation. 2 h later, reperfusion was initiated by withdrawal of the suture. The experimental timeline was illustrated in Figure 7C.

### 2.6 Laser Speckle Contrast Imaging (LSCI) and Image Processing

Laser speckle flowmetry was used to detect the cortical blood flow changes observed through the cranial window as described previously (Dunn et al. 2001). A 785 nm laser diode (Thorlabs, USA) was used to diffusely illuminate the cortical surface through the thinned skull. A Charge‐coupled device (CCD) camera (Basler 602F, Basler Vision Technologies, Ahrensburg, Germany) attached to a stereomicroscope (Nikon SMZ 1000, Nikon) and custom‐developed software (courtesy of A.K. Dunn) was used for LSC, which were captured every 10 s during a 2‐h experiment to construct cortical blood flow maps. A baseline image was constructed by averaging 10 consecutive images before ischemia for each saved inverse correlation time (ICT) image sequence. The field of view was adjusted using a variable magnification objective on the microscope (Nikon SMZ 1000, Nikon). Drift in the z‐axis was inevitable due to the blood volume changes in the ischemic tissue. To compare the rCBF changes induced by MCAo in selected regions of cortex, LSC images were later imported to ImageJ v1.42q NIH as image sequences and then saved in TIFF format for further analysis. To transform contrast values of each image within the saved sequence to rCBF values, inverse correlation time images were obtained with MATLAB (Mathworks) software by taking the camera exposure time (5 ms) into consideration. Subsequent ICT images were differentially divided by the baseline image to yield the relative CBF values, and then all calculated values for each pixel were averaged separately for the duration of ischemia. The results were constructed as a single image and pseudo‐colored to illustrate the average percent flow change for each pixel during ischemia (ΔCBFisch). Comparing rCBF changes between DAB and saline, GYS1^Nestin‐KO^ and WT mice, two ROIs depicting ischemic core and penumbra within the MCA territory were selected for each experiment from areas devoid of pial vessels. The mean and standard errors of each ROI from the ischemic core and peri‐infarct area were calculated, and histograms were generated with MATLAB software.

### 2.7 Non‐Invasive Systolic Blood Pressure Measurement

Besides rCBF assessment, systolic blood pressure was measured from the tail via laser‐speckle flowmetry. A blood pressure cuff (ADInstruments) was placed to the proximal part of the tail, and this cuff was inflated periodically through a non‐invasive blood pressure (NIBP ML125, ADInstruments) controller. Peanut oil was applied to the tail to enhance the reflectance. LSC images were captured every 1 s during the tail cuff inflation for a minute. A baseline image was constructed by averaging 10 consecutive images before inflating the cuff. LSC images were later imported to ImageJ v1.42q NIH as image sequences and then saved in TIFF format for further analysis. Two ROIs depicting both tail veins and the artery were selected for each experiment, and the graphs regarding blood flow measurements from the tail vessels were constructed. For each measurement, the graphs plotting the tail cuff pressure and tail blood flow values were superimposed. Systolic blood pressure values were obtained by measuring the pressure values at the points where the blood flow was interrupted and restored back. The measurements were performed 2 times 10 min before and 120 min after MCAo experiments.

### 2.8 Immunofluorescence Studies

Mice were sacrificed via transcardial perfusion with 4% paraformaldehyde (PFA) solution (dissolved in phosphate‐buffered saline (PBS)) under a lethal dose of chloral hydrate (Sigma‐Aldrich, # 05–2650, 500 mg/kg intraperitoneally). Harvested brain tissues were post‐fixated in PFA solution for 24 h and then placed in 30% sucrose (in PBS) at 4°C for at least 2 days to obtain tissue cryopreservation. Brains were cut via a sliding microtome (Leica SM2000R) with a thickness of 50 μm. To permeabilize, the sections were incubated in TBS containing 0.3% Triton‐X for 30 min at room temperature, then 10 mM sodium citrate (pH: 6.0) for 30 min, heated to 80°C for antigen retrieval. The sections were blocked in 1% bovine serum albumin containing 0.3% Triton‐X, 10% normal goat serum (NGS) (0.3% TBS‐T/1% BSA, 10% NGS) solution for 1 h at room temperature and then incubated with primary antibodies in 0.3% TBS‐T overnight at +4°C. After rinsing, the sections were then incubated with the respective secondary antibodies in 0.3% TBS‐T for 90 min at room temperature. After rinsing the sections three times for 10 min with TBS, sections were then incubated with ‘Fluorescein’ labeled *Lycopersicon esculentum* Lectin (dissolved in Tris‐buffered saline (TBS); Vector Laboratories, Burlingame, CA) at +4°C overnight to visualize vessels. Finally, the sections were mounted and covered with anti‐fade reagent containing 1/1000 Hoechst 33258 (Molecular Probes, ThermoFisher Scientific) to label cellular nuclei. Images of the stained sections were obtained with a Leica SP8 laser‐scanning confocal microscope equipped with a diode laser 488 nm and 552 nm source for fluorescence illumination with a X‐, Y‐, and Z‐movement controller, and a high‐resolution PMT (Zeiss, Oberkochen, Germany) and HyD (Leica) detectors.

Pericytes were identified using a convergent approach based on three established mural cell markers (CD13, PDGFR‐β, and NG2), each evaluated in separate staining experiments. As these markers may label contiguous populations of mural cells and limit single‐cell resolution (Grant et al. 2019), pericyte localization was assigned specifically at sites where positive marker labeling co‐localized with a clear morphological constriction of the lectin‐labeled capillary. Primary antibodies were obtained from the following sources and used at 1:200 dilutions: anti‐glycogen antibodies ESG1A9 and IV58B6 (courtesy of Dr. Hitoshi Ashida and Dr. Otto Baba), CD13 (Acris Antibodies GmBH, Cat. No: AM26636AF‐N), NG2 (Millipore, Cat. No: AB5320, Research Resource Identifier (RRID): AB_91789), and PDGFR‐β (Abcam, Cat. No: ab69506, RRID: AB_1269704) for pericytes, MCT1 (Proteintech, Cat. No: 20139‐1‐AP, AB_2878645), MCT12 (Proteintech, Cat. No: 20553‐1‐AP, RRID: AB_10693621), Hexokinase‐2 (HK2) (Proteintech, Cat. No: 22029‐1‐AP, RRID: AB_11182717), and GPR81 (Novus Biologicals, Cat. No: NLS2095, RRID: AB_10001932). Secondary antibodies were obtained from the following sources and used at 1:200 dilutions: Alexa Fluor 488 conjugated goat anti‐Mouse IgM (Jackson ImmunoResearch, Cat. No: 715‐545‐140, RRID: AB_2340845) for anti‐glycogen antibodies, Alexa Fluor 488 conjugated Goat anti‐Rabbit IgG (H&L) (ThermoFisher Scientific, Cat. No: A‐11008), Alexa Fluor 594 conjugated Goat anti‐Rabbit IgG (H&L) (ThermoFisher Scientific, Cat. No: A‐11012), Alexa Fluor 647 AffiniPure Donkey Anti‐Chicken IgY (IgG) (H + L) (Jackson ImmunoResearch, Cat. No: 703‐605‐155, RRID: AB_2340379), and Alexa Fluor 568 AffiniPure Donkey Anti‐Rat IgG (H + L) (Jackson ImmunoResearch, Cat. No: 712‐575‐153, RRID: AB_3095476).

Antibody specificity was first assessed using standard negative controls, including omission of the primary antibody. For non‐commercial glycogen detection methods (PAS‐based staining with aldehyde blocking and IV58B6 immunostaining), specificity was further supported by complementary genetic (GYS1 knockout) and pharmacological (DAB) approaches, which produced the expected directionally consistent changes in signal intensity. Immunostainings for metabolic transporters, including GPR81, were used as supportive descriptive readouts and interpreted based on reproducible spatial patterns and their relationship to CD13‐positive microvascular structures rather than as primary quantitative endpoints.

### 2.9 Western Blotting

Mice were sacrificed via rapid decapitation under a lethal dose of chloral hydrate (Sigma‐Aldrich, Cat. No: 05‐2650, 500 mg/kg intraperitoneally). The entire brain of 3‐ to 6‐month‐old mice was quickly frozen in liquid nitrogen. Protein was solubilized by probe sonication in RIPA buffer (30% w/v) with protease and phosphatase inhibitors for 2 h at 4°C. 50 μg of brain lysates were run on a 4%–12% SDS‐PAGE gel using MOPS running buffer, then transferred to PVDF membranes overnight at 4°C via wet transfer method. Blots were blocked with 5% bovine serum albumin in TBS‐Tween‐20 (0.1%) for 1 h at room temperature, then incubated with previously characterized primary antibodies: CD13 (Acris Antibodies GmBH, Cat. No: AM26636AF‐N; 1:1000), NG2 (Millipore, Cat. No: AB5320, RRID: AB_91789; 1:1000), and PDGFR‐β (Abcam, Cat. No: ab69506, RRID: AB_1269704; 1:1000), GYS‐1 (Cell Signaling Technologies, Cat. No: 3893, RRID: AB_2279563; 1:1000). HRP‐conjugated secondary antibodies (Goat anti‐Rabbit IgG (H + L) Cross‐Adsorbed Secondary Antibody, HRP, Cat. No: G‐21234, RRID: AB_1500696; 1:5000, Goat anti‐Mouse IgG (H + L) Cross‐Adsorbed Secondary Antibody, HRP, Cat. No: G‐21040, RRID: AB_2536527; 1:5000) were incubated for 90 min at room temperature and detected on an Odyssey Infrared scanner using chemiluminescent reagent (SuperSignal West Femto Maximum Sensitivity Substrate, ThermoFisher Scientific). Each signal was normalized to Beta‐actin (ThermoFisher Scientific, Cat. No: PA1‐183; RRID: AB_2539914; 1:7500).

### 2.10 Ex Vivo Stereological Analysis of Cerebral CD13‐Positive Pericyte Induced Microvessel Constrictions, Microvessel Branching, and Quantification

Stereological studies were carried out in brain sections to evaluate and quantify the CD13‐positive pericyte‐associated constrictions in microvessels objectively (Alarcon‐Martinez et al. 2019). 10 out of 100 coronal brain sections with a thickness of 50 μm, which were taken 500 μm apart spanning the middle cerebral artery (MCA) territory (from the first 1 mm to 6 mm, 5 mm anteroposteriorly in total), were obtained from each brain. To span the MCA territory in an unbiased way, coronal sections were obtained with sliding microtome cutting thickness (50 μm) through 5 mm anteroposteriorly starting from the 1st mm to the 6th. Thus, one out of every ten serial sections was analyzed. In each brain section, for each hemisphere, 10 dissectors were examined using a three‐dimensional disector frame (field of view: 240 × 160 μm; area per field of view: 0.0384 mm^2^; sampled thickness along the *z*‐axis: 40 μm). Across all sampled fields (*n* = 100 per hemisphere), this resulted in a total evaluated area of 3.84 m^2^. Density values were calculated based on counts per mm^2^ and were therefore independent of the total sampled area. CD13‐positive pericyte constrictions were quantified within the three‐dimensional disectors across the 40 μm sampled thickness. Vessels, pericytes, and nuclei were marked with Lectin, anti‐CD13, and Hoecsht 33 258 (Molecular Probes, ThermoFisher Scientific), respectively. We quantified the number of CD13‐positive pericyte induced microvessel constrictions under 40× magnification, defined as a focal narrowing which is more than 20% of the diameter of the upstream and/or downstream vessel segment, and analyzed whether these constrictions colocalized with pericytes (< 10 μm away from the pericyte soma). Microvascular constrictions were counted among the capillaries having their diameters less than 9 μm, and any constrictions that were closer than a respective distance for a pericyte soma (< 10 μm) were not quantified. The constriction counts were expressed in constrictions/mm^2^. Microvessel branching points were manually counted and expressed in mm^2^.

### 2.11 PAS Histochemistry and Lectin Staining to Assess Peri‐Microvascular Glycogen Levels

In order to compute the peri‐microvascular glycogen, brain sections were labeled for glycogen using periodic acid‐Schiff (PAS) staining (Gurer et al. 2009) and vessels were marked by Lectin. 50 μm thick brain sections were permeabilized with Tris buffered saline (TBS) containing 0.3% triton‐X for 30 min at room temperature, then kept in a solution of 10 mM sodium citrate (pH: 6.0) for 30 min heated to 80°C for antigen retrieval. Sections were oxidized in Periodic acid solution (0.5% in double deionized water (ddH_2_0), pH: 7.4) for 10 min at room temperature. Since Schiff's agent also reacts with non‐glycogen aldehyde groups, the sections were kept in dimedone solution (pH: 7.4) at 60°C for 20 min. Sections were then incubated with Schiff's agent for 15 min at room temperature and the PAS reaction was fixed by heating ddH_2_O for 5 min. Lastly, sections were incubated with TBS containing Lectin at +4°C overnight. Sections were imaged by a fluorescent microscope (400×, Eclipse E600, Nikon Instruments Inc., Melville, NY) equipped with a manually controlled specimen stage for X, Y, and Z‐axis, a color camera (model DXM1200, Nikon Instruments Inc.), fluorescent light source (HB‐10104AF, Nikon Instruments Inc.), and an image analysis software (NIS‐Elements, Version 3.22, Nikon Instruments Inc.). 10 brain sections for each experiment were analyzed using the dissector technique, taking 4–6 dissectors per section (360 × 240 μm) covering the MCA territory (starting 1 mm from the frontal pole to 6 mm of the brain, totally 5 mm anteroposteriorly). A semiautomatic computer routine was used to identify microvessels in the fluorescent channel, and then, in the bright‐field channel, quantification of glycogen levels was performed by calculating the mean brightness over the selected microvessels, as established previously (Alarcon‐Martinez et al. 2019; Gurler et al. 2023). To make comparisons between the sections possible, each peri‐microvascular mean bright field intensity was proportioned to the average background brightfield intensity. Then, correlations among peri‐microvascular glycogen levels and total number of microvascular constrictions were analyzed. For this analysis, the amount of glycogen around microvessel walls in either DAB‐injected or ischemic mice was proportioned to that of controls. Mean brightness intensity values were assigned on a semi‐quantitative scale (Alarcon‐Martinez et al. 2019; Gurler et al. 2023) ranging from 0 to 6 in increments of 1 in DAB‐injected ones, 0 to 1 in increments of 0.2 in ischemic mice.

### 2.12 Quantification of Adjusted Ischemic Infarct Volume

Coronal brain sections processed with Nissl staining were evaluated under a phase contrast microscope. The ischemic area was delineated and measured by using NIS Elements AR v4.2 software. Adjusted infarct area was calculated by subtracting the non‐infarcted area of the ipsilateral hemisphere from the contralateral hemisphere, and infarct volume was calculated by multiplying the sum of infarct areas of sequential coronal sections 500 μm apart by the total anteroposterior distance of 5 mm (Swanson et al. 1990).

### 2.13 Statistical Analysis

All data were analyzed using the GraphPad Prism statistical analysis program. All photographs were prepared with the same microscope under identical illumination and data capture conditions. Both the people taking photographs, recording LSCI, and analyzing the data were blinded to the experimental and treatment conditions. All summary data are expressed using the superplot style as median and interquartile range (Q1–Q3). All data sets were tested for normality using the Shapiro–Wilk normality test. The remaining non‐normally distributed data were analyzed using the Mann–Whitney *U* test (for two groups), Kruskal‐Wallis (for more than two groups), or the “Wilcoxon signed rank” test (for two dependent groups). The Kruskal‐Wallis and Mann–Whitney *U* tests were used to compare regional cerebral blood flow (rCBF) changes, microvessel constriction counts, microvascular branching, total peri‐microvascular glycogen‐related PAS/IV58B6 intensity, ischemic volumes, microvascular monocarboxylate transporter‐1 coverage, and systolic blood pressure measurements. The Wilcoxon signed‐rank test was used for systolic blood pressure values within each experiment. Similar variance was assured for all groups, which were statistically compared. Differences with a *p* < 0.05 were statistically significant. No test for outliers was conducted. The power analysis for the sample size was performed using G*Power 3.1 with a power of 90% and an alpha level of 0.05. Effect size estimation was based on prior experimental data on variation in semi‐stereological assessment of the capillary constriction values.

### 2.14 Data and Code Availability

All summarized data are in the main text, figures, or supporting information. Primary data (e.g., images, spreadsheets) and the original MATLAB code for spatiotemporal regional cerebral blood flow (rCBF) measurements via pseudo‐colored maps are available upon request from the lead contact. Preprint of this manuscript was uploaded on bioRxiv; August 26, 2022; https://www.biorxiv.org/content/10.1101/2022.08.24.505172v1.

**Table:** 

Reagent or Resource	Source	Identifier
**Antibodies**		
*Lycopersicon esculentum* (Tomato) Lectin (LEL, TL), DyLight 488	Vector Labs	Cat. No:Cat. No: DL‐1174‐1, RRID: N/A
Aminopeptidase N/CD13 Antibody	Acris Antibodies GmBH	Cat. No:Cat. No: AM26636AF‐N, RRID: N/A
NG2	Millipore	Cat. No:Cat. No: AB5320, RRID: AB_91789
PDGFR‐β	Abcam	Cat. No:Cat. No: ab69506, RRID: AB_1269704
GYS‐1	Cell Signaling Technologies	Cat. No:Cat. No: 3893, RRID: AB_2279563
Beta‐Actin	ThermoFisher Scientific	Cat. No: PA1‐183, RRID: AB_2608409
GFAP	Abcam	Cat. No: ab4674, RRID: AB_304558
MCT1 Polyclonal antibody	Proteintech	Cat. No: 20139‐1‐AP, RRID: AB_2878645
MCT12 Polyclonal antibody	Proteintech	Cat. No: 20553‐1‐AP, RRID: AB_10693621
Hexokinase 2 Polyclonal antibody	Proteintech	Cat. No: 22029‐1‐AP, RRID: AB_11182717
GPR81 Antibody	Novus Biologicals	Cat. No: NLS2095, RRID: AB_10001932
IV58B6	courtesy of Dr. Otto Baba	N/A
ESG1A9	courtesy of Dr. Hitoshi Ashida	N/A
AffiniPure F(ab’)_2_ Fragment Donkey Anti‐Mouse IgM	Jackson Immunoresearch	Cat. No: 715‐006‐020, RRID: AB_2340760
Alexa Fluor 488 AffiniPure Donkey Anti‐Mouse IgM	Jackson Immunoresearch	Cat. No: 715‐545‐140, RRID: AB_2340845
Alexa Fluor 488 conjugated Goat anti‐Rabbit IgG (H&L)	ThermoFisher Scientific	Cat. No: A‐11008, RRID: N/A
Alexa Fluor 594 conjugated Goat anti‐Rabbit IgG (H&L)	ThermoFisher Scientific	Cat. No: A‐11012, RRID: N/A
Alexa Fluor 647 AffiniPure Donkey Anti‐Chicken IgY (IgG) (H + L)	Jackson Immunoresearch	Cat. No: 703‐605‐155, RRID: AB_2340379
Alexa Fluor 568 AffiniPure Donkey Anti‐Rat IgG (H + L)	Jackson Immunoresearch	Cat. No: 712‐575‐153, RRID: AB_3095476
Goat anti‐Mouse IgG (H + L) Cross‐Adsorbed Secondary Antibody, HRP	ThermoFisher Scientific	Cat. No: G‐21040, RRID: AB_2536527
Goat anti‐Rabbit IgG (H + L) Cross‐Adsorbed Secondary Antibody, HRP	ThermoFisher Scientific	Cat. No: G‐21234, RRID: AB_1500696
**Chemicals, peptides, and recombinant proteins**		
DAB (1,4‐dideoksi‐1,4‐imino‐d‐arabinitol hydrochloride)	Sigma‐Aldrich	Cat. No: D1542
Sodium D‐lactate	Sigma‐Aldrich	Cat. No: 71716
Sodium L‐lactate	Sigma‐Aldrich	Cat. No: 71718
Chloral hydrate	Sigma‐Aldrich	Cat. No: 05–2650
Periodic acid	Sigma‐Aldrich	Cat. No: 375810
Schiff's reagent	Merck	Cat. No: 3952016
Dimedone (5,5‐Dimethyl‐1,3‐cyclohexanedione)	Sigma‐Aldrich	Cat. No: D153303
TRIS‐buffered saline (TBS, 10X) pH 7.4	ThermoFisher	Cat. No: J60764.K2
RIPA Lysis and Extraction Buffer	Sigma‐Aldrich	Cat. No: 89900
Triton‐X 100	Sigma‐Aldrich	Cat. No: X100
Tween‐20	Sigma‐Aldrich	Cat. No: P1379
Halt Protease and Phosphatase Inhibitor Cocktail (100X)	ThermoFisher	Cat. No: 78440
NuPAGE Bis‐Tris Mini Protein Gels, 4%–12%, 1.0–1.5 mm	ThermoFisher	Cat. No: NP0322BOX
NuPAGE MOPS SDS Running Buffer (20X)	ThermoFisher	Cat. No: NP0001
PVDF Transfer Membranes	ThermoFisher	Cat. No: 88518
VECTASHIELD PLUS Antifade Mounting Medium with DAPI (H‐2000)	Vector Labs	Cat. No: H‐2000, RRID: N/A
**Experimental models: Organisms/strains**		
Swiss Albino mice	Jackson Laboratories	Strain #:034608
		RRID:IMSR_JAX:034608
C57Bl/6 wild‐type (WT) mice	Jackson Laboratories	Strain: #:000664
		RRID: IMSR_JAX:000664
GYS1^Nestin‐KO^ mice	Courtesy of Dr. Duran and Dr. Guinovart	N/A
GYS1^Gfap‐KO^ mice		
**Software and algorithms**		
ImageJ/Fiji	NIH	https://imagej.net/software/fiji/
Prism‐ GraphPad	Dotmatics	https://www.graphpad.com/scientific‐software/prism/www.graphpad.com/scientific‐software/prism/
Endnote 20	Clarivate	https://endnote.com/?language=en
Adobe Photoshop	Adobe	https://www.adobe.com/products/photoshop.html/
Adobe Illustrator	Adobe	https://www.adobe.com/products/illustrator.html/

## 3 Results

### 3.1 Disrupted Glycogen Utilization Compromises Cerebral Blood Flow

**FIGURE 1:**
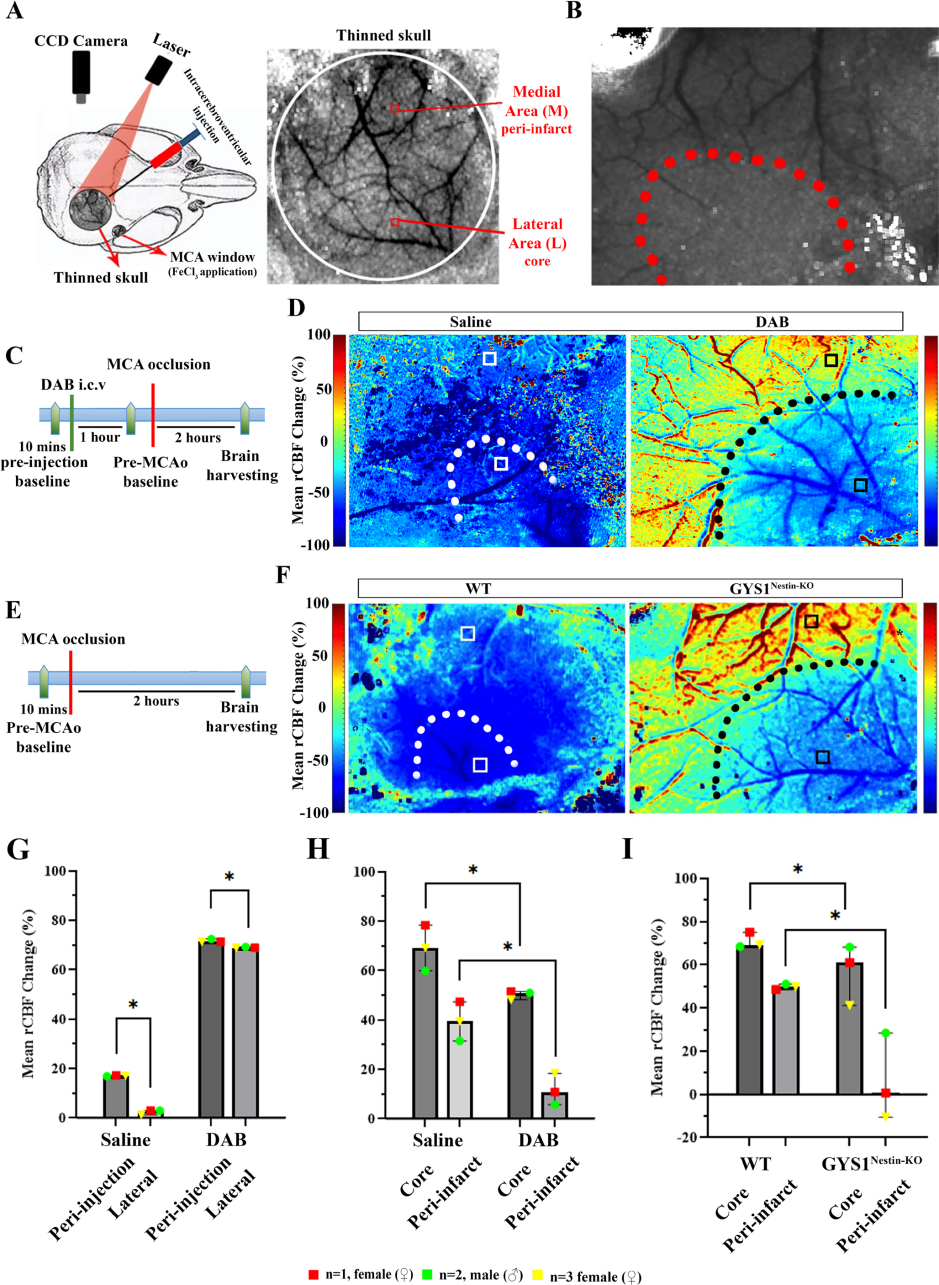
Glycogen utilization affects cortical regional Cerebral Blood Flow (rCBF) dynamics after successful Middle Cerebral Artery occlusion (MCAo). Laser speckle contrast imaging (LSCI) is used to detect the cortical blood flow changes observed through the cranial window, as described previously (Dunn et al. 2001). (A) Schematic illustration of experimental setup (left panel). DAB and saline injections via intracerebroventricularly (i.c.v.) are also performed through the cranial window. Middle cerebral artery occlusion (MCAo) is introduced through another cranial window via ferric chloride (FeCl_3_) administration for 10 min. Charge‐coupled device (CCD) camera captures the reflection of laser‐illuminated cortical region through thinned skull, and then regional cerebral blood flow (rCBF) changes are observed throughout the whole experiments as described. The visualization of the cortical surface is demonstrated on the right panel. Vessels appear as black, whereas parenchyma is gray. The medial (depicting peri‐infarct area) and lateral (ischemic core) region of interests (ROI) are carefully chosen to avoid high caliber vasculature. (B) Representative image taken during MCAo. Red dots delineate the area of ischemic core after successful clotting of the middle cerebral artery (MCA). (C) Schematic illustration of the experimental flow in Saline and DAB injections and MCAo afterwards. LSCI imaging was initiated 10 min before the i.c.v. injection was performed. At one hour of i.c.v. injection, MCAo was performed, and images were being taken until 5 min before the mice were sacrificed. (D) Pseudo‐colored maps are created after MCAo of saline (left panel) and DAB (right panel) injected WT mice. Every LSCI image taken 10 s apart proportioned to baseline after i.c.v. injections (described in Methods). Scale bars on the right and left sides show that cold colors represent a decrease (0 through ‐%100) whereas hot colors, an increase in rCBF from baseline (0 through +%100). The quantifications of mean rCBF change are performed in white (saline) and black (DAB) boxes from medial (peri‐infarct) and lateral (core) ROIs. White (saline) and black (DAB) dots describe the MCA core territories, respectively. Drift in the z axis (vessel shift) was inevitable because of the blood volume changes in the ischemic tissue. (E) Schematic illustration of the experimental flow in MCAo of WT and GYS1^Nestin‐KO^ mice. LSCI imaging was initiated 10 min before MCAo was performed and images were being taken until 5 min before the mice were sacrificed for 2 h. (F) rCBF alterations are also assessed in GYS1^Nestin‐KO^ mice (right panel) during permanent MCAo compared to WT (left panel) via pseudo‐colored maps. The representations are the same as above. GYS1^Nestin‐KO^ mice display a similar rCBF alteration pattern (right panel) as DAB‐injected counterparts (right panel at C). (G) Quantifications of mean rCBF change in each ROI after saline or DAB injections. Results are given as percentages (*n* = 3 animals per group). The average values of mean rCBF changes measured at medial (M) and lateral (L) regions of interest (white and black boxes) are shown as gray after i.c.v. saline or DAB injections, red after i.c.v. injections. Data represented as median and interquartile range (Q1‐Q3). Red circle: *N* = 1, female (♀), Green square: *N* = 2, male (♂), Yellow triangle: *N* = 3, female (♀). Two‐way analysis of Mann–Whitney *U*, *n* = 3; *p < 0.05. (H) Ipsilateral MCAo for 1 h, decreased mean rCBF further in the ischemic core of DAB injected mice: However, lower than vehicle‐injected controls (Two‐way analysis of Mann–Whitney U, *n* = 3; *p < 0.05). In DAB‐injected ischemic mice, peri‐infarct ROI displays less reduction of mean rCBF compared to vehicle‐injected ischemic controls (Two‐way analysis of Mann–Whitney *U*, *n* = 3; *p < 0.05). Data represented as median and interquartile range (Q1–Q3). Red circle: *N* = 1, female (♀), Green square: *N* = 2, male (♂), Yellow triangle: *N* = 3, female (♀). (I) Quantifications of mean rCBF change in each ROI in MCAo of WT and GYS1^Nestin‐KO^ mice. Results are given as percentages (*n* = 3 animals per group). The average values of mean rCBF changes measured at medial (M) and lateral (L) regions of interest (white and black boxes) are shown as red and black after MCAo, respectively. Ischemia caused a decrease in rCBF in the ischemic core of both WT and GYS1^Nestin‐KO^ mice, but the decrease was less in the GYS1^Nestin‐KO^ group (Two‐way analysis of Mann–Whitney *U*, *n* = 3; *p < 0.05). Besides, the mean decrease was also significantly lower in the peri‐infarct area in wild‐type mice, compared to GYS1^Nestin‐KO^ (Two‐way analysis of Kruskal‐Wallis and Mann–Whitney *U*, *n* = 3; *p < 0.05). Data represented as median and interquartile range (Q1–Q3). Red circle: *N* = 1, female (♀), Green square: *N* = 2, male (♂), Yellow triangle: *N* = 3, female (♀).

To investigate the functional consequences of disrupted utilization of peri‐microvascular glycogen and CD13‐positive pericyte‐associated microvascular constrictions under physiological and pathological conditions in the brain, we studied regional cortical cerebral blood flow (rCBF) changes (Figure 1). Without compromising blood flow changes via surgical intervention, we performed Laser Speckle Contrast Imaging (LSCI) in thinned skulls of mice (Figure 1A,B). Representative image of LSCI overviewing how brain parenchyma and vasculature look after middle cerebral artery occlusion (MCAo) was shown in Figure 1B. Schematic illustration of the experimental paradigm in intracerebroventricular (i.c.v.) injection and MCAo explained the timing of each intervention under LSCI (Figure 1C). LSCI was performed throughout ipsilateral permanent MCAo before, during and 1 h after ipsilateral permanent MCAo in i.c.v. injection and transgenic groups (Figure 1C–E). rCBF alteration maps were created by the semi‐automatic analysis routine (MATLAB‐Simulink) quantifying the contrast changes throughout the experiment compared to basal conditions as established before in our lab (Figure 1D–F, details in Methods, (Donmez‐Demir et al. 2023)).

DAB resulted in a robust reduction of mean rCBF (medial: 71.35% ± 1.17%, lateral: 68.87% ± 0.39%) compared to vehicle injection (medial: 17.03% ± 0.27%, lateral: 2.95% ± 1.65%, *n* = 3, Mann–Whitney *U* test, *U* = 0, *p* = 0.025) within an hour (Figure 1G). Ipsilateral MCAo for 1‐h decreased mean rCBF further in the ischemic core of DAB‐injected mice (Figure 1H, 50.98% ± 3.34%); however, lower than vehicle‐injected controls (69.22% ± 18.58%, *p* = 0.025, Mann–Whitney *U* test, *U* = 0, p = 0.025) (Figure 1H). In DAB‐injected ischemic mice, the peri‐infarct area displayed less reduction of mean rCBF (10.89% ± 12.79%) compared to vehicle‐injected ischemic controls (39.45% ± 15.82%, Mann–Whitney *U* test, *U* = 0, *p* = 0.025) (Figure 1H), possibly because of the maximum detectable reduction of rCBF after DAB.

rCBF alterations were also assessed in GYS1^Nestin‐KO^ mice during ipsilateral permanent MCAo compared to wild‐type controls via LSCI (Figure 1I). Ischemia induced a decrease in rCBF in the ischemic core of both wild type (69.29% ± 6.72%, *n* = 3) and GYS1^Nestin‐KO^ (60.98% ± 27.07%, *n* = 3) mice, but the percentage decrease was less in the GYS1^Nestin‐KO^ group (Mann–Whitney *U* test, *U* = 0, *p* = 0.025) (Figure 1I). Besides, the mean decrease was also significantly lower in the peri‐infarct area in wild‐type mice (49.94% ± 2.45%), compared to GYS1^Nestin‐KO^ (0.65% ± 38.96%, Mann–Whitney *U* test, *U* = 0, *p* = 0.025) (Figure 1I) in accordance with DAB and ischemia results. These results suggested that GYS1^Nestin‐KO^ mice may have a lower basal rCBF level, demonstrating less rCBF decrease after ischemia. Representative graphs depicting rCBF throughout each experiment are shown in Figure S1.

As the physiological parameters of mice have a notable effect on the outcome measures such as hemodynamic differences and infarct volume, it was highly critical to standardize parameters like systolic blood pressure in the peri‐experimental setting; hence, it was closely monitored (Table 1). Systolic blood pressure was measured by using a tail‐cuff non‐invasively via LSCI to eliminate the probable hemodynamic disturbances due to inevitable blood loss in conventional invasive measurements. No significant changes were observed between the mean systolic blood pressure values measured before and after the permanent MCAo among groups (Table 1).

**TABLE 1:** Non‐invasive blood pressure measurement during MCAo via tail‐cuff method.

Systolic blood pressure (mmHg)	Saline	DAB	WT	GYS1^NestinKO^	P‐value
Before experiment	100.1 ± 3.9	99.2 ± 4.5	110.3 ± 4.3	105.7 ± 1.5	0.376
End of the experiment	96.7 ± 7.6	95.4 ± 7.6	106.0 ± 1.9	101.7 ± 5.2	0.5
Overall	98.4 ± 3.9	100.2 ± 3.3	108.2 ± 2.9	103.7 ± 2.6	0.17

### 3.2 Disruption of CNS Glycogen Utilization Causes CD13‐Positive Pericyte‐Mediated Microvascular Constrictions in the Mouse Brain

To investigate the disruption of glycogen utilization, pharmacological and genetic methods were used, and resultant microvascular impairment was assessed in 10 coronal brain sections in 10 areas separated by a minimum of 500 μm in each hemisphere by unbiased semi‐stereological quantification parameters as established before in our lab (Figure S2A) (Alarcon‐Martinez et al. 2019). To show this impairment, microvascular constrictions were assessed by *Lycopersicon esculentum* Lectin and CD13+ pericytes (Figure S2B). Given the existence of multiple established markers used to identify pericytes, we performed immunofluorescence studies using PDGFR‐β, NG2, and CD13. We observed that CD13 and NG2 labeled microvascular constrictions in a pattern similar to lectin, whereas a subset of constrictions lacked PDGFR‐β labeling (Figure S2C). While CD13 and NG2 labeling consistently overlapped with lectin‐positive constriction sites, a subset of these constrictions lacked detectable PDGFR‐β signal. Based on its robust and consistent labeling across all constriction sites, CD13 was therefore used as the primary mural cell marker for subsequent analyses. To address the possibility that transcardial perfusion might deplete glycogen and thereby influence the occurrence of microvascular constrictions, we additionally assessed the microvascular tree in flash‐frozen sections (Figure S2D). No significant differences were observed between the two methods, and all subsequent analyses were therefore performed on transcardially perfusion‐fixed tissues.

As the pharmacological approach, a potent glycogen phosphorylase inhibitor, 1,4‐Dideoxy‐1,4‐imino‐D‐Arabinitol hydrochloride (DAB) was administered intracerebroventricularly (i.c.v.) in naïve wild‐type mice. The effective DAB dose (0.25 M) was decided based on a previous study (Kilic et al. 2018). As the half‐life of DAB was found to be 6 h in in vitro enzyme inhibition studies (Walls et al. 2008), and there were limited data regarding its in vivo effects, several time points were examined after i.c.v. DAB injection. To define the possible effects that might be caused by i.c.v. injection or tissue processing, another group of mice received i.c.v. sterile saline (the dissolvent of DAB) as vehicle (*n* = 3) and all data were presented with superplots.

**FIGURE 2:**
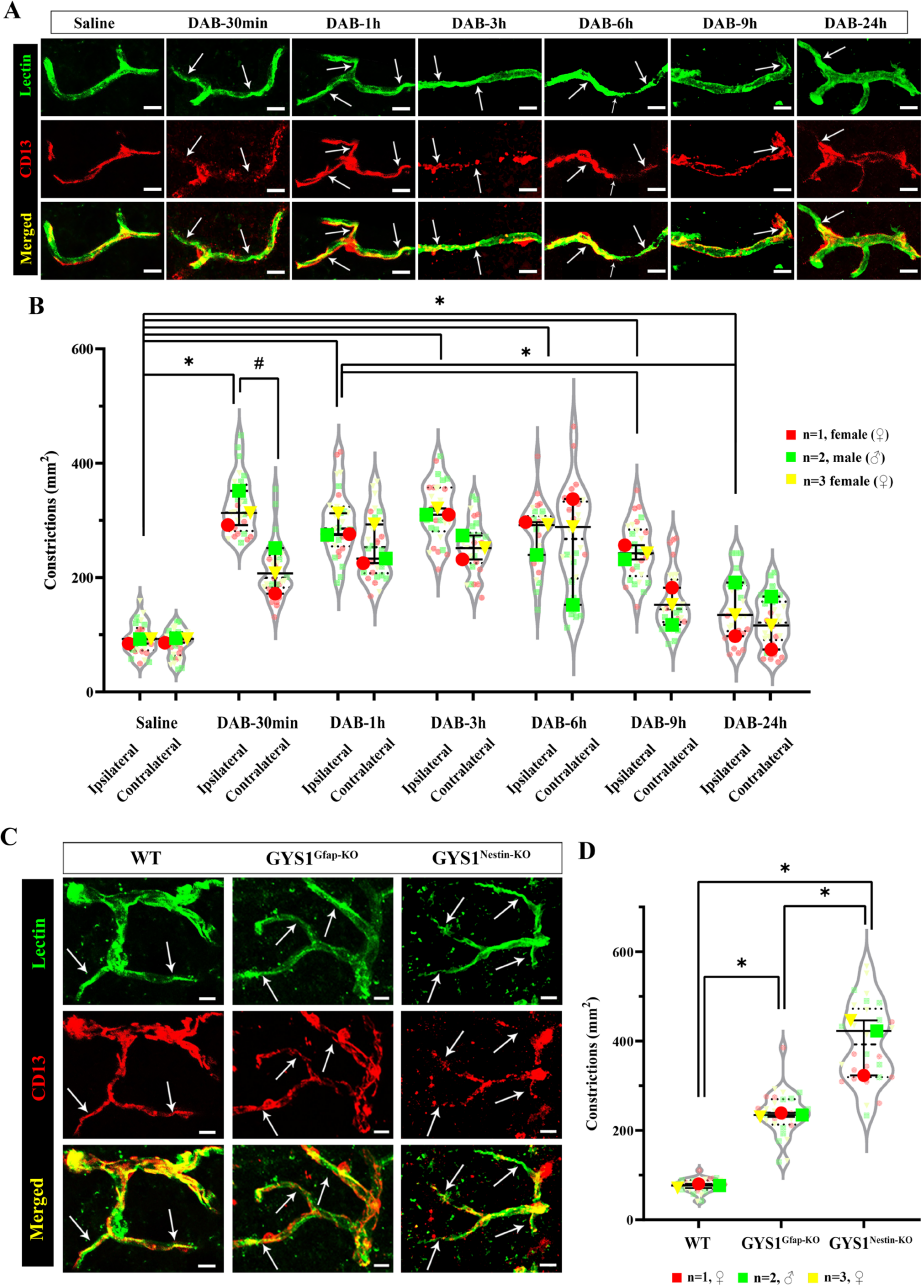
Disruption of CNS glycogen utilization results in CD13 positive (CD13+) capillary pericyte‐mediated constrictions. CD13‐positive capillary pericyte‐associated constrictions were evaluated in Swiss albino, wild‐type (WT), GYS1^Gfap‐KO^, and GYS1^Nestin‐KO^ mice. (A) Experiments performed in adult Swiss albino male and female mice intracerebroventricularly (i.c.v) administered with saline (vehicle) or DAB (as indicated in figure) sacrificed after 30 min, 1 h, 3 h, 6 h, 9 h, and 24 h (shown left to right, respectively). *Lycopersicon esculentum* Lectin (Lectin) (upper panel‐green) and CD13 (middle panel‐red) double labeling (merged as lower panel) reveals increased microvascular constrictions 30 min after DAB injections, shown by arrows. Images represent a 3D reconstruction of 40‐μm z‐stack. Scale bars, 10 μm. (B) Microvascular constrictions in the ipsilateral and contralateral hemispheres were quantified semi‐stereologically. Constriction counts were demonstrated as superplots, which displayed counts from each coronal section individually with different colors as shown in the right‐hand legend. DAB (i.c.v) injections result in a robust increase of constrictions after 30 min, persisting for 6 h. Although the number of constrictions started to decline after 9 h, the impact of DAB on these constrictions is not fully reversible even after 24 h. Quantification of CD13+ pericyte‐associated microvascular constrictions in ten fields for each hemisphere spanning the middle cerebral artery (MCA) territory (240 μm × 160 μm) per animal (*n* = 3 animals per group, described in Methods). Two‐way analysis of Kruskal‐Wallis and Mann–Whitney *U*, *n* = 3; *p < 0.05. Red circle: *N* = 1, female (♀), Green square: *N* = 2, male (♂), Yellow triangle: *N* = 3, female (♀). Data represented as median and interquartile range (Q1–Q3). (C) Adult naïve wild‐type (WT), GYS1^Nestin‐KO^ and GYS1^Gfap‐KO^ mice are sacrificed, and brain sections are labeled with Lectin (upper panel‐green) and CD13 (middle panel‐red and merged as lower panel). GYS1^Nestin‐KO^ mice demonstrate the highest number of microvascular constrictions as shown by arrows. Images represent 3D reconstruction of 40‐μm z‐stack. Scale bars, 10 μm. (D) CD13+ pericyte‐associated microvascular constrictions in adult naïve wild‐type (WT), GYS1^Nestin‐KO^ and GYS1^Gfap‐KO^ mice are quantified. Transgenic mice have a higher number of constrictions. Quantification of CD13+ pericyte‐associated microvascular constrictions in ten fields for each hemisphere spanning MCA territory (240 × 160 μm) per animal (*n* = 3 animals per group). Mann–Whitney *U*, *n* = 3; *p < 0.05. Data was shown as violin‐plot/superplots with individual values. Data represented as median and interquartile range (Q1–Q3). Red circle: *N* = 1, female (♀), Green square: *N* = 2, male (♂), Yellow triangle: *N* = 3, female (♀).

Mice treated with i.c.v. DAB were sacrificed after 30‐min, 1‐h, 3‐h, 6‐h, 9‐h, and 24‐h (*n* = 3 for all time points). Respective images of microvasculature and pericyte constrictions from each time were shown as Figure 2A. The constrictive effect of DAB injection, counted as the mean number of microvascular constrictions (number/mm^2^) was significantly higher than i.c.v. vehicle injections at 30‐min to 6‐h of DAB administration (Kruskal Wallis test, *H* = 34.32, *p* = 0.0011) (Figure 2B). However, microvascular constrictions started to diminish after 9‐h (and 24‐h), probably due to the reversible inhibition of glycogen phosphorylase DAB (Figure 2B). Thus, it was demonstrated that in vivo pharmacological inhibition of glycogen phosphorylase resulted in a significant increase in CD13‐positive pericyte constrictions of brain microvessels in a reversible and time‐dependent manner.

Next, to determine the direct effect of peri‐microvascular glycogen, we used GYS1^Gfap‐KO^ and GYS1^Nestin‐KO^ mice. In GYS1^Gfap‐KO^ and GYS1^Nestin‐KO^ mice, the enzyme glycogen synthase‐1, responsible for the production of glycogen, was selectively deleted from the astrocytes and brain respectively (Duran and Guinovart 2015; Duran et al. 2013; Saez et al. 2014). CD13‐positive pericyte‐associated microvessel constrictions (number/mm^2^) were shown with a white arrow as in Figure 2C. Naïve GYS1^Gfap‐KO^ (236.3 ± 56.0, *n* = 3) and GYS1^Nestin‐KO^ (387.5 ± 151.5, *n* = 3) mice displayed significantly higher microvascular constrictions when compared to wild type naïve mice (76.79 ± 20.76, *n* = 3) (Kruskal Wallis test, *H* = 7.20, *p* = 0.0036) (Figure 2C,D).

Genetic glycogen depletion models revealed alterations in microvasculature as well as the constrictions (Figure S2E,F). Western blotting and immunofluorescence showed that both GYS1^Gfap‐KO^ and GYS1^Nestin‐KO^ mice reduced GYS‐1 expression in the brain. PDGFR‐β expression was reduced in GYS1^Nestin‐KO^ mice whereas CD13 and NG2 were comparable to WT controls (Figure S2E, Figure S7). Moreover, we wanted to assess glycogen stores in GYS1^Nestin‐KO^ mice. PAS and IV58B6 immunostaining demonstrated no significant glycogen in the brain (Figure S2G,H). Lastly, we quantified the microvascular branching to show if these developmental changes affected the capillary tree. Total number of capillary branches revealed a loss of microvascular coverage in GYS1^Nestin‐KO^ but not GYS1^Gfap‐KO^ (Figure S3A,B). Accordingly, conclusions regarding the astrocyte–pericyte lactate shuttle are primarily drawn from the GYS1^Gfap‐KO^ model, which does not exhibit baseline alterations in microvascular coverage. Findings from the GYS1^Nestin‐KO^ mice are interpreted as reflecting the broader impact of developmental glycogen depletion on microvascular structure and vulnerability, rather than serving as the primary test of astrocyte‐specific mechanisms. GYS1^Nestin‐KO^ mice were nevertheless included in subsequent experiments to assess whether a broad, developmental loss of brain glycogen recapitulates the microvascular phenotype induced by pharmacological inhibition of glycogenolysis (DAB).

### 3.3 Disrupted Peri‐Microvascular Glycogen Increases Susceptibility to Ischemia

**FIGURE 3:**
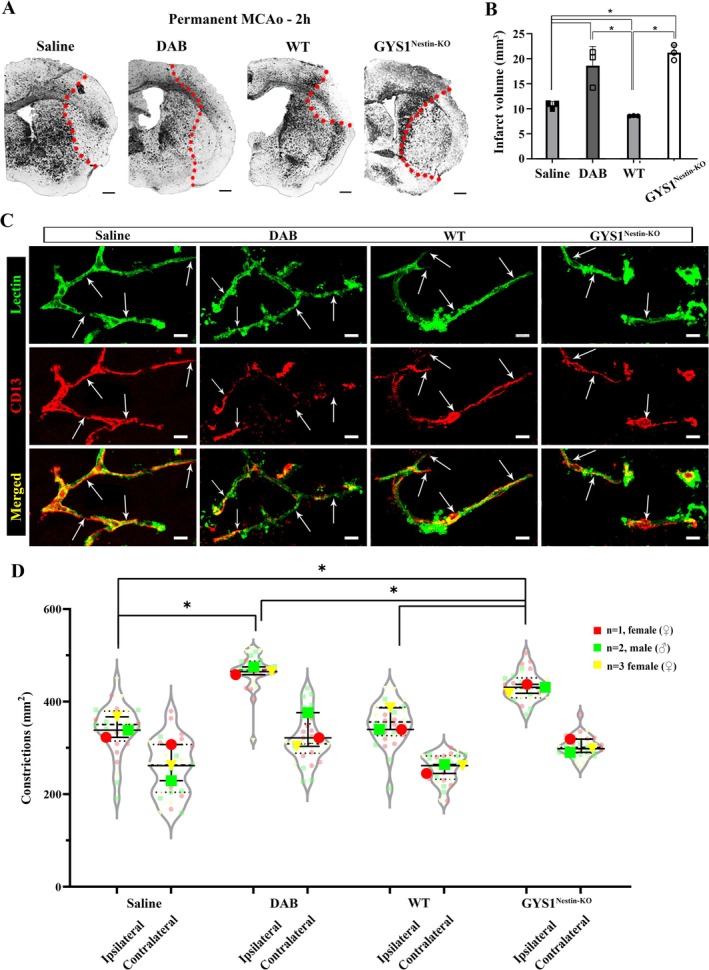
Susceptibility to cerebral ischemia is potentiated via disruption of CNS glycogen utilization. Permanent middle cerebral artery occlusion (MCAo) experiments were performed in Saline, DAB‐injected WT, naïve WT, and GYS1^Nestin‐KO^ mice (*n* = 3 animals per group). (A) Representative images taken from Cresyl violet‐stained sections of ischemic mice via phase contrast microscopy (as indicated in the figure) that underwent 2‐h MCAo. Red dots are placed over the border between core and peri‐infarct areas. Scale bars, 500 μm. (B) Infarct volumes after 2‐h MCAo are measured and then quantified with volume correction (Swanson et al. 1990). Ischemic infarct volumes after MCAo were significantly higher in DAB‐treated mice than in saline‐treated groups (Two‐way analysis of Mann–Whitney *U*, *n* = 3; *p < 0.05). Infarct volumes of the GYS1^Nestin‐KO^ group were also higher when compared to wild type (Two‐way analysis of Mann–Whitney *U*, *n* = 3; *p < 0.05). Data represented as median and interquartile range (Q1–Q3). (C) *Lycopersicon esculentum* Lectin (upper panel‐green) and CD13 (middle panel‐red) double labeling (merged as lower panel) reveals increased microvascular constrictions (arrows) 2 h after MCAo in Saline, DAB injected WT, naïve WT and GYS1^Nestin‐KO^ mice (left to right, respectively). Images represent 3D reconstruction of 40‐μm z‐stack. Scale bars, 10 μm. (D) Quantification of microvascular constrictions after MCAo in Saline, DAB injected WT, naïve WT and GYS1^Nestin‐KO^ mice (*n* = 3 animals per group). Quantification of CD13+ pericyte‐associated microvascular constrictions. Ischemia further resulted in a higher number of microvascular constrictions in peri‐microvascular glycogen‐disrupted mice (DAB‐injected and GYS1^Nestin‐KO^) compared to ischemic controls (Two‐way analysis of Mann–Whitney *U*, *n* = 3; *p < 0.05). Data was shown as violin‐plot/superplots with individual values. Data represented as median and interquartile range (Q1–Q3). Red circle: *N* = 1, female (♀), Green square: *N* = 2, male (♂), Yellow triangle: *N* = 3, female (♀).

Since disrupted glycogen utilization led to cerebral blood flow compromise via microvascular constrictions, we assessed ischemic infarct volumes after 2 h of middle cerebral artery occlusion (MCAo) in pharmacological and genetic models (Figure 3A). Ischemic infarct volumes 2‐h after MCAo were significantly higher in DAB‐treated mice (20.29 ± 7.113.85) than vehicle‐treated groups (11.11 ± 1.3 mm^3^) (*n* = 3/groups) (Kruskal Wallis test, *H* = 9.462, *p* = 0.0014). Infarct volumes of the GYS1^Nestin‐KO^ group were also higher (21.22 ± 3.03) when compared to the wild‐type (8.58 ± 0.09 mm^3^) (*n* = 3/groups) (, Mann–Whitney *U* test, *U* = 0, *p* = 0.025) (Figure 3A,B). This increase in infarct volume further pointed out the vulnerability of mice with disrupted peri‐microvascular glycogen to ischemia. Accordingly, DAB injected mice (464.80 ± 17.00/mm^2^) and GYS1^Nestin‐KO^ (431.00 ± 19.50/mm^2^) had prevalent CD13‐positive pericyte‐associated microvascular constrictions in the ischemic area (Figure 3C,D) compared to vehicle injected (338.5 ± 44.3) and wild type mice (339.8 ± 46.90, *n* = 3/groups, Mann–Whitney *U* test, *U* = 0, *p* = 0.025) (Figure 3C,D). Ischemia further resulted in a higher number of microvascular constrictions in mice with disrupted glycogen (DAB‐injected and GYS1^Nestin‐KO^) compared to ischemic controls (Kruskal Wallis test, *H* = 20.81, *p* = 0.0041). We should also note that DAB‐injected group demonstrated a slightly higher number of constrictions compared to GYS1^Nestin‐KO^ mice after ischemia. This is because most of the vessel segments in GYS1^Nestin‐KO^ mice collapsed, which couldn't be quantified as constrictions (Figure S3). Overall, disruption of glycogen utilization induced further susceptibility during ischemia.

### 3.4 Glycogen Phosphorylase Inhibition Disrupts Glycogen Utilization Where CD13‐Positive Pericyte‐Mediated Microvascular Constrictions Occur

**FIGURE 4:**
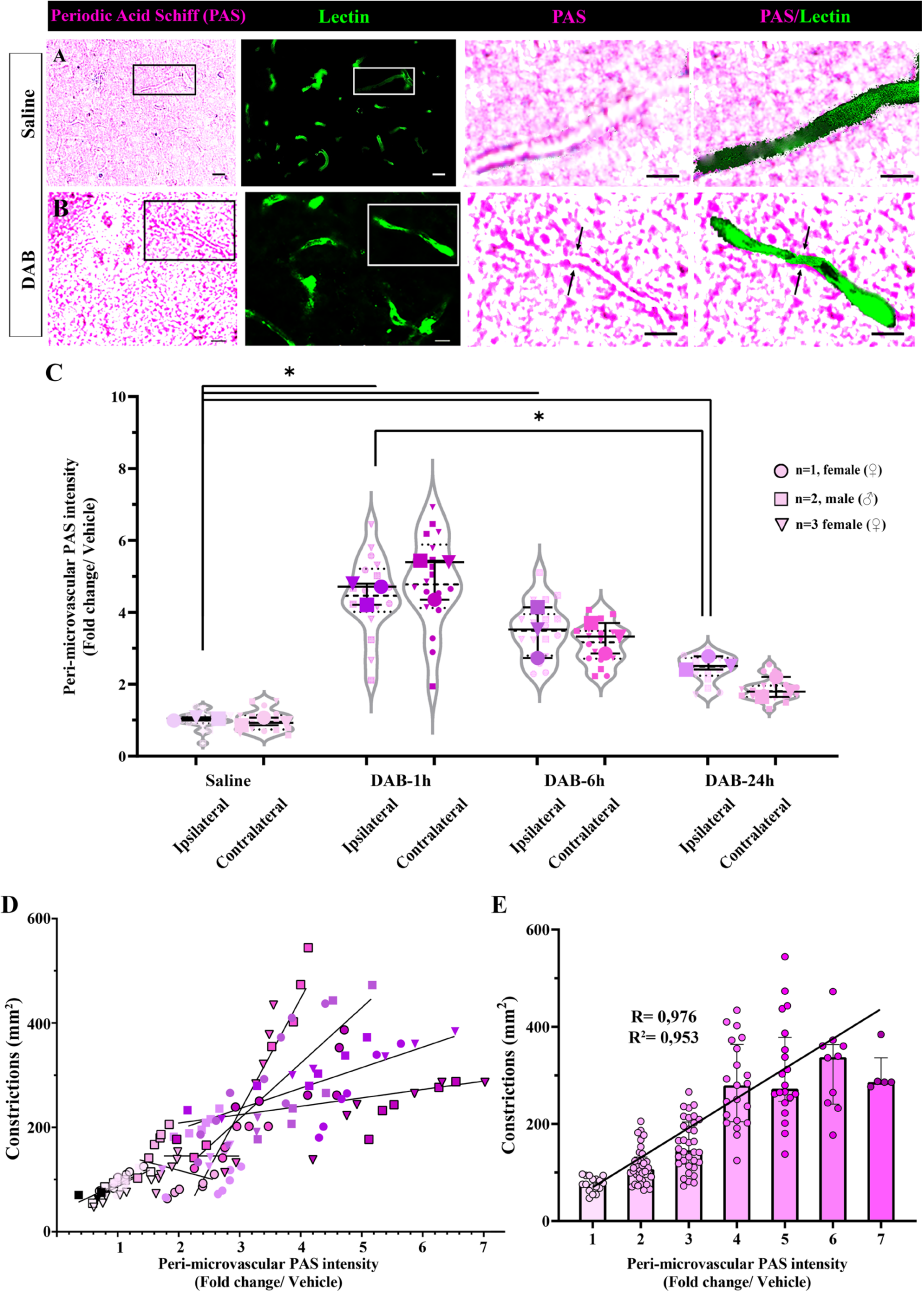
Intracerebroventricularly administered glycogen phosphorylase inhibitor 1,4‐Dideoxy‐1,4‐imino‐D‐arabinitol hydrochloride (DAB) causes increased peri‐microvascular glycogen and correlates with microvascular constrictions. Periodic acid Schiff (PAS)‐Lycopersicon *esculentum* Lectin (Lectin) double staining is performed in intracerebroventricular (i.c.v) saline or DAB‐injected Swiss albino mice to show glycogen around microvessels in the tissue. (A) PAS (left panel) and Lectin (second from the left) stained sections from saline‐injected mice reveal stored glycogen around microvessels (black and white box). The areas delineated via boxes are displayed with higher magnification, and these images show peri‐microvascular glycogen (third and fourth panels from the left). Scale bars: 20 μm (left and second to left), 10 μm (third and fourth to left). (B) DAB injection causes an increase in glycogen deposition around microvessels (left panel‐black box), coinciding with microvessel constrictions (second from the left‐white box). Magnified areas display increased glycogen‐related PAS intensity especially around microvascular constrictions (arrows). Scale bars: 20 μm (left and second to left), 10 μm (third and fourth to left). (C) A semi‐automatic software macro (NIS Elements 4.3) is used to quantify the peri‐microvascular PAS intensity (Alarcon‐Martinez et al. 2019). The peri‐microvascular PAS intensity is measured in both hemispheres after injections. DAB‐treated brains (*n* = 3 animals per group) displayed a significant increase in the PAS intensity compared to saline (Two‐way analysis of Mann–Whitney *U*, *n* = 3; *p < 0.05). Circle: *N* = 1, female (♀), Square: *N* = 2, male (♂), Triangle: *N* = 3, female (♀). Data are shown as violin‐plot/super plots with individual values. (D) Total number of microvascular constrictions and respective peri‐microvascular PAS intensity are plotted. Each line indicates the linear regression results in each ipsilateral and contralateral hemisphere of vehicle and DAB‐injected groups. (E) The total number of constrictions (*y*‐axis) is positively correlated to the mean peri‐microvascular glycogen levels (*x*‐axis). Hence glycogen is there but cannot be utilized by glycogen phosphorylase. Pearson's correlation, *R* = 0.976, *R* ^2^ = 0.953. Data represented as median and interquartile range (Q1–Q3).

Periodic acid Schiff (PAS) staining was used as it is the gold standard for showing glycogen in the tissue. Using an aldehyde blocker, dimedone, allowed us to visualize only glycogen, but not the glycoproteins and proteoglycans present in the tissue (Bulmer 1959). Thereafter, a semi‐automatic computer software (’Macro’, NIS Elements 4.3) that was previously established in our laboratory was used to quantify the peri‐microvascular glycogen (Alarcon‐Martinez et al. 2019; Gurler et al. 2023). In this method, the peri‐microvascular PAS signal intensity was measured using Lectin as a mask (Figure 4A,B) and normalized to vehicle‐injected controls. DAB treated brains displayed a significant increase in peri‐microvascular PAS intensity (Figure 4C) within 1‐h (4.71 ± 0.58 fold), 6‐h (3.52 ± 1.41 fold), and 24‐h (2.51 ± 0.37 fold) compared to vehicle‐treated group (*n* = 3/group, 1.04 ± 0.09 fold, Kruskal Wallis test, *H* = 21.87, *p* = 0.0027) (Figure 4C). The increase coincided with the location of microvascular constrictions; hence glycogen is present there, but possibly because of the disrupted glycogen utilization in the peri‐microvascular astrocyte processes, cannot be utilized by glycogen phosphorylase.

To further investigate the relationship between glycogen utilization and CD13‐positive pericyte‐associated microvessel constrictions, correlations among peri‐microvascular PAS intensity and total number of microvascular constrictions were analyzed. Linear regression analyses between PAS intensity change around microvessels and respective constrictions were performed. Each time point after DAB injection showed that peri‐microvascular PAS intensity was positively correlated with the number of constrictions (Figure 4D). Also, we pooled and categorized all data points from each DAB injection to demonstrate the glycogen‐constriction relationship independent from the groups. DAB‐induced pericyte‐associated constrictions tightly correlated with higher glycogen levels (Figure 4E, Pearson's correlation, *R* = 0.976, *R* ^2^ = 0.953, *p* = 0.0001). Therefore, we showed that glycogen phosphorylase inhibition disrupted glycogen utilization and exacerbated CD13‐positive pericyte‐associated microvascular constrictions.

### 3.5 Cerebral Ischemia Depletes Peri‐Microvascular Glycogen and Further Increases DAB Related CD13‐Positive Pericyte‐Mediated Microvascular Constrictions

**FIGURE 5:**
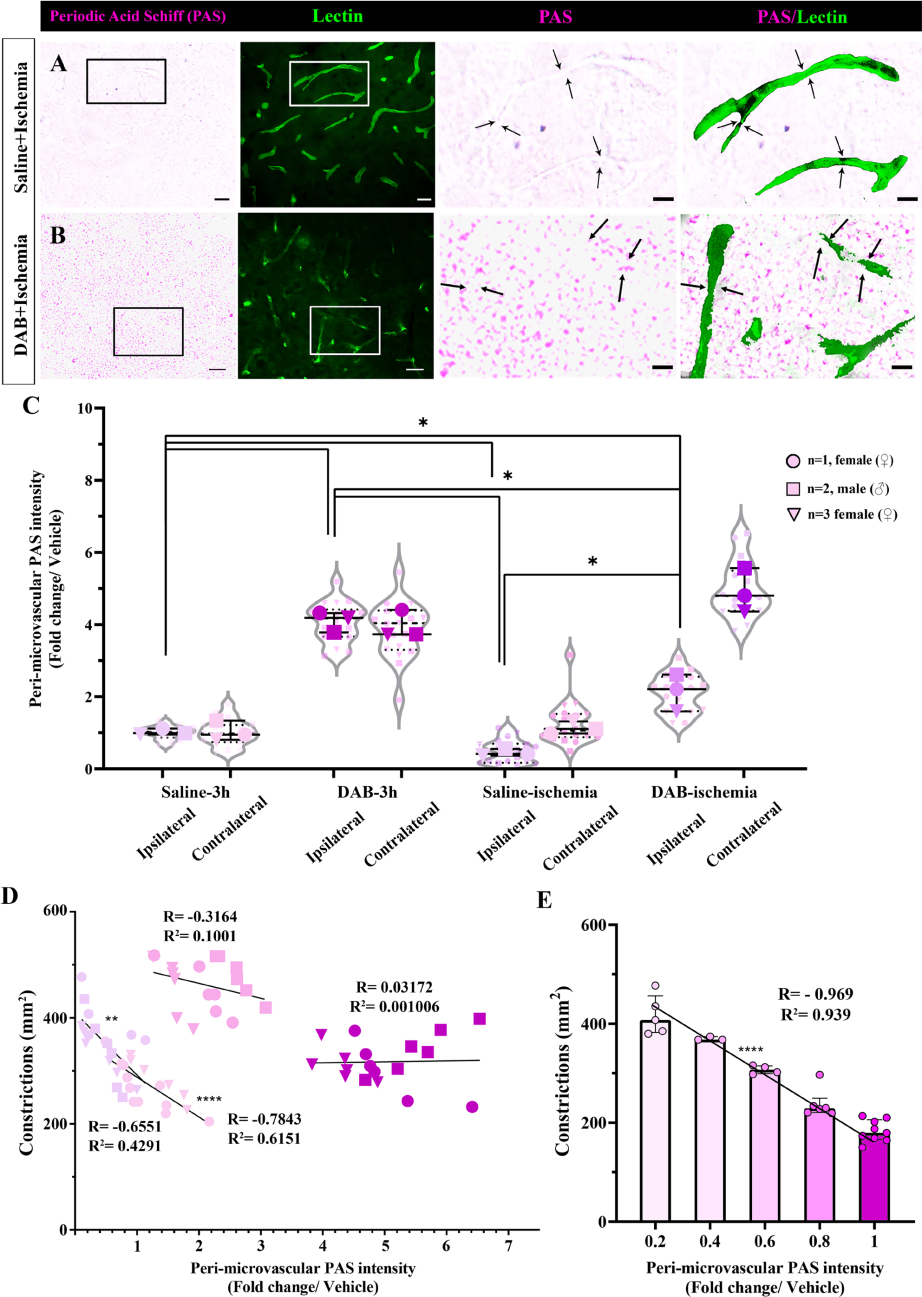
Cerebral ischemia depletes peri‐microvascular glycogen and increases microvascular constrictions. Periodic acid Schiff (PAS)‐ *Lycopersicon esculentum* Lectin (Lectin) double staining are performed in Swiss albino mice which underwent middle cerebral artery occlusion (MCAo) after intracerebroventricular (i.c.v) saline or DAB injections (*n* = 3 animals per group). (A) PAS (left panel) and Lectin (second from the left) stained sections from saline‐injected ischemic mice reveal depletion of stored glycogen around microvessels (black and white box). The areas delineated via boxes are displayed with higher magnification and these images show diminished peri‐microvascular glycogen (third and fourth panels from the left) coinciding the constrictions (arrows). Scale bars; 20 μm (left and second to left), 10 μm (third and fourth to left). (B) In mice subjected to 2‐h permanent MCAo after DAB injection (*n* = 3), there is an increase in peri‐microvascular glycogen (left panel‐black box) coinciding with a higher number of microvessel constrictions (second from the left‐white box). Magnified areas display increased glycogen‐related PAS intensity, especially around microvascular constrictions (arrows). Scale bars; 20 μm (left and second to left), 10 μm (third and fourth to left). (C) Quantification of mean peri‐microvascular PAS intensity via a semi‐automatic software macro (NIS Elements 4.3) is used (Alarcon‐Martinez et al., Acta Neuropath. Comm. 2019). Peri‐microvascular PAS intensity of DAB‐treated brains (*n* = 3 animals per group) displayed a significant increase compared to saline (Two‐way analysis of Mann–Whitney *U*, *n* = 3; *p < 0.05). Circle: *N* = 1, female (♀), Square: *N* = 2, male (♂), Triangle: *N* = 3, female (♀). Data was shown as violin‐plot/superplots with individual values. Data represented as median and interquartile range (Q1‐Q3). (D) Total number of microvascular constrictions and respective peri‐microvascular PAS intensity are plotted. Each line indicates the linear regression results in each ipsilateral and contralateral hemisphere of the vehicle and DAB‐injected groups after ischemia. Pearson's correlation, *R* = −0.6551, *R* ^2^ = 0.4291 in ipsilateral cortex, *R* = −0.7843, *R* ^2^ = 0.6151 in contralateral cortex. (E) The total number of constrictions (*y*‐axis) are negatively correlated to the peri‐microvascular PAS intensity (*x*‐axis). Although DAB blocks glycogen utilization, ischemia depletes and causes a higher number of microvascular constrictions. Pearson's correlation, *R* = −0.969, *R* ^2^ = 0.939. Data represented as median and interquartile range (Q1–Q3).

To evaluate the effect of cerebral ischemia on glycogen utilization, 1 h after i.c.v. vehicle (saline) injection, permanent middle cerebral artery occlusion (MCAo) was performed for 2‐h (*n* = 3). A decrease in PAS intensity (very light pink color), depicting glycogen depletion, was observed throughout the MCA territory corresponding to the infarction (Figure 5A–C) (0.42 ± 0.19‐fold), and was significantly lower when compared to the non‐ischemic vehicle injections (0.99 ± 0.17, *n* = 3, Kruskal Wallis test, H = 21.29, *p* = 0.0034). We also induced 2‐h permanent MCAo after DAB injection (*n* = 3). As previously shown, peri‐microvascular PAS intensity was higher in mice subjected to DAB (2.21 ± 1.02‐fold) compared to both non‐ischemic and ischemic vehicle‐injected ipsilateral cortex (Mann–Whitney *U* test, *U* = 0, *p* = 0.025; Kruskal‐Wallis test, *H* = 10.38, *p* < 0.0001). High peri‐microvascular PAS intensity values among the contralateral cortex in the DAB‐treated group (4.80 ± 1.20‐fold) demonstrated that ischemia caused glycogen depletion against the DAB effect. (Figure 5C).

After 2‐h of permanent ischemia in vehicle‐injected mice, the number of CD13‐positive pericyte‐associated constricted microvessels correlated with the presence of low peri‐microvascular PAS intensity (Pearson's correlation, *R* = −0.6551, *R* ^2^ = 0.4291 in ipsilateral cortex, *R* = −0.7843, *R* ^2^ = 0.6151 in contralateral cortex) (Figure 5D). When all data from the vehicle‐treated ischemic group were pooled, peri‐microvascular PAS intensity and the total number of microvascular constrictions in ischemic groups showed a significant negative correlation (Pearson's correlation, *R* = −0.969, *R* ^2^ = 0.939) (Figure 5E).

**FIGURE 6:**
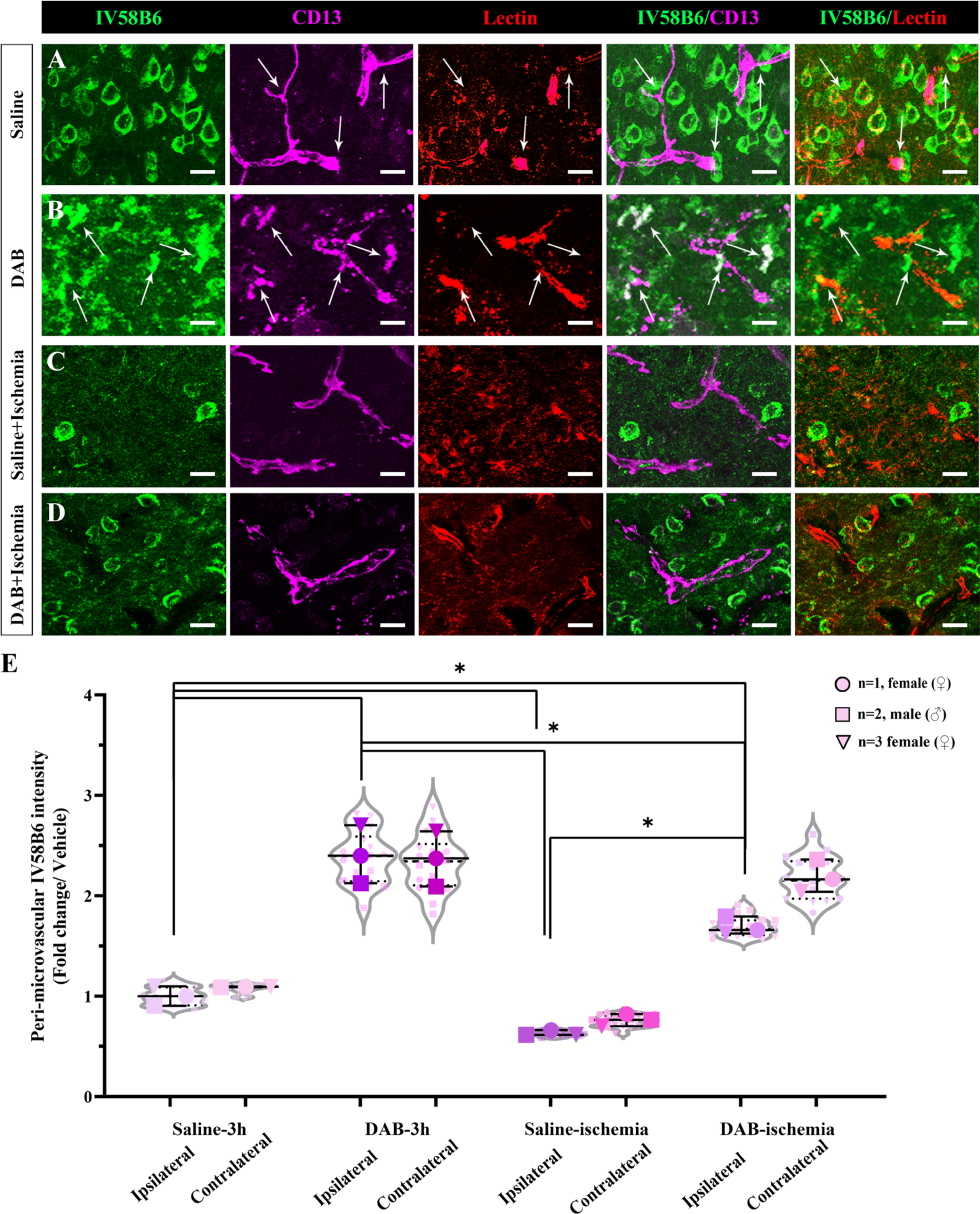
Glycogen‐specific antibody IV58B6 reveals peri‐microvascular glycogen changes after ischemia and glycogen phosphorylase inhibition. Immunofluorescence with anti‐glycogen antibody IV58B6 in naïve and ischemic mice. Triple labeling with IV58B6 (left panel‐green), CD13 (magenta) and Lectin (middle panel‐red) in (A) Saline‐injected (upper row), (B) DAB‐injected (2nd row), (C) Saline‐injected ischemic (3rd row), and (D) DAB‐injected ischemic (4th row) mice. Anti‐glycogen antibody reveals substantial deposition around CD13+ pericytes and microvessels in the merged image (right panels) of DAB‐injected mice as shown with arrows. The loss of anti‐glycogen antibody‐related signal intensity reveals the coincided nature of microvessel constrictions and glycogen depletion in both saline and DAB‐injected ischemic mice (3rd and 4th rows). Images represent 3D reconstruction of 40‐μm z‐stack. Scale bars, 10 μm. Images represent 3D reconstruction of 40‐μm z‐stack. Scale bars, 10 μm. (E) Peri‐microvascular IV58B6 intensity in fold change over vehicle (saline) treated mice in both hemispheres was quantified semi‐stereologically (*n* = 3 animals per group, described in Methods, Two‐way analysis of Kruskal‐Wallis and Mann–Whitney *U*, *n* = 3; *p < 0.05). Circle: *N* = 1, female (♀), Square: *N* = 2, male (♂), Triangle: *N* = 3, female (♀). Data was shown as violin‐plot/superplots with individual values.

In addition to PAS histochemical staining, immunohistochemical studies were performed to show stored glycogen using non‐commercial IV58B6 and ESG1A9 antibodies that label glycogen granules, thus glycogen stores. Glycogen is mostly stored in peri‐microvascular astrocyte processes (Swanson et al. 1989). Glycogen labeling, aka IV58B6 or ESG1A9‐positive granules, was evident in the GFAP‐positive astrocyte processes covering the microvascular walls, where constrictions were not present (Figure 6A; Figure S4A). IV58B6 additionally revealed GFAP‐positive cellular bodies throughout the gray matter (Figure S4A). When DAB is administered, peri‐microvascular IV58B6 glycogen drastically increased (Figure 6B) and coincided with microvascular constrictions. Following 2‐h permanent MCAo, there was a drastic reduction of peri‐microvascular glycogen in the ischemic core area (Figure 6C–E; Figure S5D). Also, glycogen labeling was absent in the ischemia‐induced microvessel constriction locations (Figure 6C–E). When ischemia was induced after DAB administration, peri‐microvascular IV58B6‐positive granules were depleted (Figure 6D,E). Like PAS, IV58B6 immunofluorescence demonstrated that ischemia overrode glycogen phosphorylase inhibition and caused glycogen depletion (Figure 6D,E, Kruskal‐Wallis test, *H* = 21.65, *p* = 0.0029).

### 3.6 L‐Lactate Administration Reversed DAB‐Induced CD13‐Positive Pericyte‐Mediated Microvascular Constrictions

**FIGURE 7:**
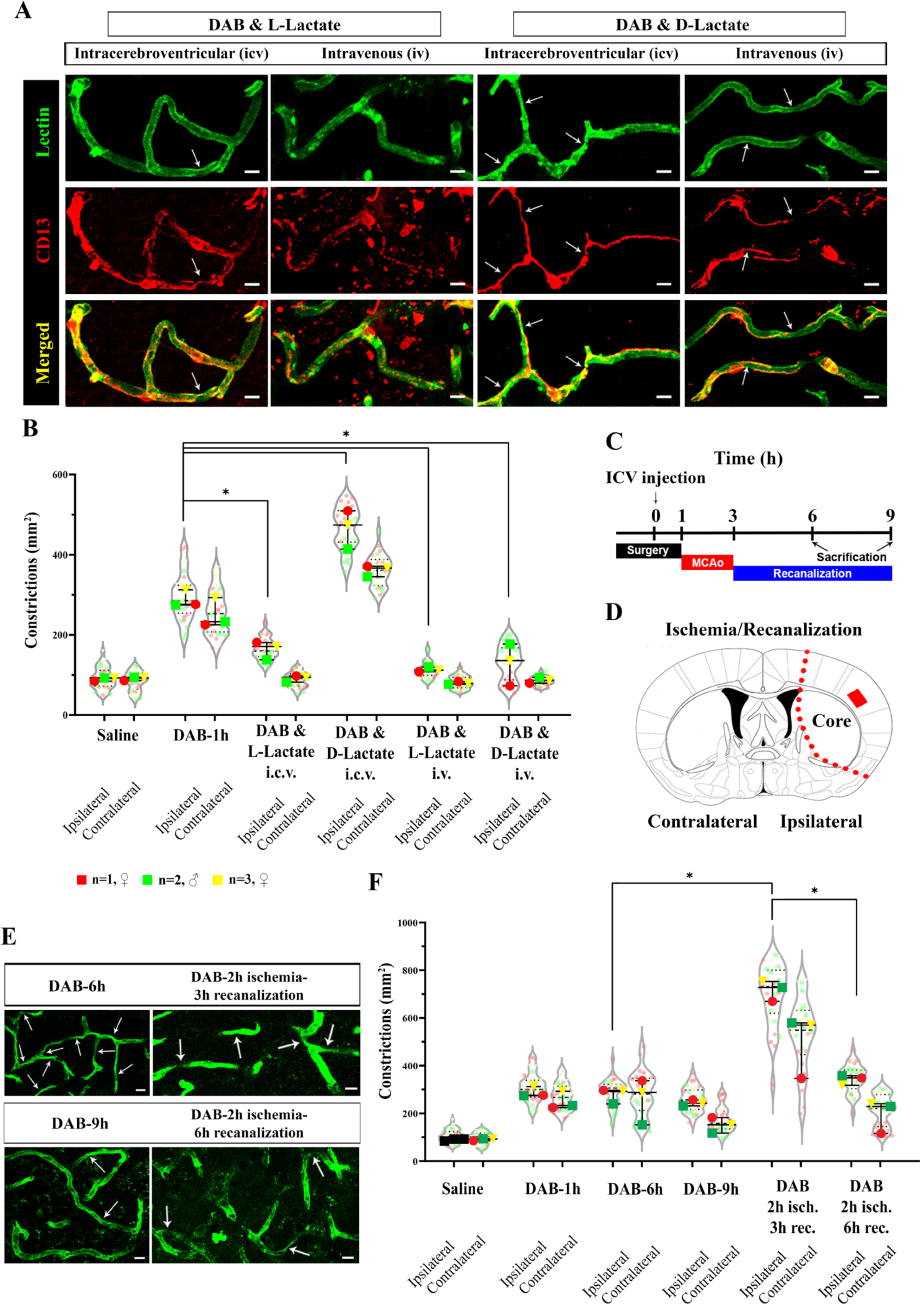
Microvascular susceptibility to disruption of CNS glycogen utilization is reversible via lactate administration and Ischemia/Recanalization (I/R). CD13‐positive capillary pericyte‐associated constrictions were evaluated in intracerebroventricular (i.c.v.) and intravenous (i.v.) DAB+ L/D‐lactate injected Swiss albino mice. (A) *Lycopersicon esculentum* Lectin (Lectin) (upper panel‐green) and CD13 (middle panel‐red) double labeling (merged as down panel) double labeling shows that L‐lactate (left panel) reverses the DAB's impact on CD13+ pericyte‐associated constrictions in both i.c.v. and i.v. administered mice. On the contrary, its enantiomer D‐Lactate (right panel) only reverses the constrictions when administered via i.v. route. Images represent 3D reconstruction of 40‐μm z‐stack. Scale bars, 10 μm. (B) Quantification of CD13+ pericyte‐associated microvascular constrictions in i.c.v. and i.v. DAB+ L/D‐lactate injected mice compared to controls (*n* = 3 animals per group, Two‐way analysis of Mann–Whitney *U*, *n* = 3; *p < 0.05). Circle: *N* = 1, female (♀), Square: *N* = 2, male (♂), Triangle: *N* = 3, female (♀). Data was shown as violin‐plot/superplots with individual values. (C) Schematic illustration for the I/R timeline. DAB i.c.v. injection preceded by proximal middle cerebral artery (MCA) occlusion (MCAo) lasting 2 h. Recanalization period extended by either 3 or 6 h, adding up to a total of 6 or 9‐h surgical interventions. (D) Schematic coronal section shown to locate where MCAo induced its effects. Red rectangle demonstrates where the representative image taken. The microvascular quantification was conducted throughout the hemispheres, and the details are mentioned in the Methods. (E) *Lycopersicon Esculentum* Lectin (Lectin) (green) labeling shows the microvascular constrictions in DAB‐6 h, DAB‐9 h, DAB‐2 h ischemia‐3 h recanalization, and DAB‐2 h ischemia‐6 h recanalization experiments. Images represent 3D reconstruction of 40‐μm z‐stack. Scale bars, 10 μm. (F) Quantification of CD13+ pericyte‐associated microvascular constrictions in i.c.v. DAB injected and I/R conducted mice compared to respective controls (*n* = 3 animals per group, Two‐way analysis of Mann–Whitney *U*, *n* = 3; *p < 0.05). Circle: *N* = 1, female (♀), Square: *N* = 2, male (♂), Triangle: N = 3, female (♀). Data was shown as violin‐plot/superplots with individual values.

Next, we tested whether CD13‐positive pericyte‐associated constrictions after DAB are reversible. Lactate is an important energy metabolite and volume transmitter transferred from astrocytes to neurons upon glycogen breakdown (Magistretti and Allaman 2015). Based on our previous studies, we used lactate as an alternative energy source to bypass disrupted glycogen utilization (Kilic et al. 2018). L‐lactate and its optical isomer D‐lactate were also administered i.c.v. and i.v. along with DAB (Figure 7A). L‐lactate was able to reverse the DAB‐induced CD13‐positive pericyte‐associated constrictions in both i.c.v. (ipsilateral: 171.00/mm^2^ ± 43.00, contralateral: 93.75 ± 15.63/mm^2^) and i.v. (ipsilateral: 112.20/mm^2^ ± 11.70, contralateral: 78.65 ± 6.51/mm^2^) administered groups. However, i.c.v. D‐lactate (ipsilateral: 474.30 ± 95.00/mm^2^, contralateral: 366.50 ± 26.00/mm^2^) (Figure 7A,B) didn't alleviate the deleterious effect of DAB on microvascular constrictions when administered i.c.v. (*n* = 3, Mann–Whitney *U* test, *U* = 0, *p* = 0.025). Strikingly, i.v. D‐lactate (ipsilateral: 136.00 ± 104.18/mm^2^, contralateral: 86.87 ± 14.32/mm^2^) recovered DAB‐induced microvascular constrictions (Figure 7A,B, Kruskal‐Wallis test, *H* = 30.81, *p* = 0.0012).

### 3.7 Simultaneous Glycogen Phosphorylase (GP) Inhibition and Ischemia/Recanalization (I/R)

Moreover, Ischemia/recanalization (I/R) models were applied in mice which cerebral glycogen utilization with DAB was inhibited (Figure 7C). Microvascular constrictions were examined after recanalization with the proximal MCA I/R model (Figure 7D–F). Microvascular constrictions with 2 h of ischemia and 3 h of reperfusion 1 h after DAB injected mice (ipsilateral hemisphere: 727.9 ± 83.3/mm^2^, contralateral hemisphere: 570.3 ± 233.0, Kruskal‐Wallis test, *H* = 30.81, *p* = 0.0012) increased significantly compared to both the 1st and 6th hours of DAB injection (*n* = 3, Mann–Whitney *U* test, *U* = 0, *p* = 0.025). Moreover, 2 h ischemia and 6‐h reperfusion, 1 h after DAB injection (349.0 ± 40.4/mm^2^) caused a greater number of microvascular constrictions in the ipsilateral hemisphere than in the 9th hour of DAB injection (*n* = 3, Mann–Whitney *U* test, *U* = 0, *p* = 0.025). However, when the recanalization duration was increased from 3 to 6 h, the effects of ischemia on microvascular constrictions also decreased dramatically (*n* = 3, Mann–Whitney *U* test, *U* = 0, *p* = 0.025). This issue can be explained by the alleviation of glycogen phosphorylase inhibition due to its reversible nature and hence the lessening impact of DAB injection on microvessel constrictions between 6th and 9th hours (*n* = 3, Mann–Whitney *U* test, *U* = 0, *p* = 0.025) (Figure 7F). Therefore, this data indicates that glycogen utilization and glycogen‐derived metabolites such as lactate may be the key which determines the pericyte contractility thus the patency of microvasculature pointing out the possibility to overcome the irreversible microvascular constrictions and no‐reflow.

### 3.8 Glycogen Regulates Lactate Transporters at the Neurogliovascular Level

**FIGURE 8:**
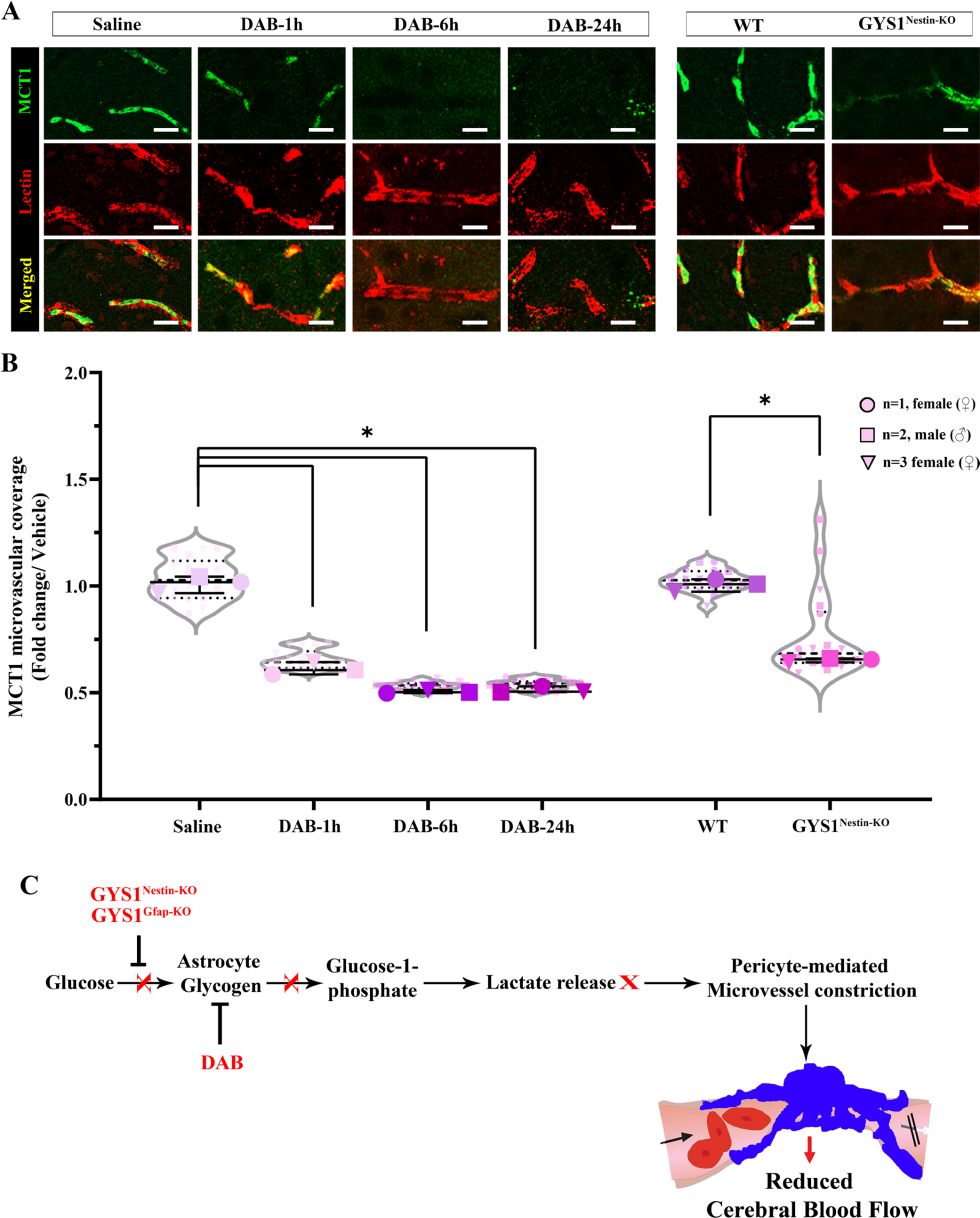
Endothelial lactate transporter MCT‐1 (Monocarboxylate transporter‐1) coverage reduces with disrupted glycogen metabolism. Monocarboxylate transporter‐1 (MCT‐1)‐labeled capillary coverage was evaluated in Swiss albino, wild‐type (WT), and GYS1^Nestin‐KO^ mice. (A) Experiments performed in adult Swiss albino male and female mice intracerebroventricularly (i.c.v) administered saline (vehicle) or DAB (as indicated in figure) sacrificed after 1, 6, and 24 h (shown left to right, respectively). MCT‐1 (upper panel‐green) and *Lycopersicon esculentum* Lectin (Lectin) (middle panel‐red) double labeling (merged as lower panel) reveals decreased microvascular coverage of MCT‐1 1 h after DAB injections and persisted for at least 24 h. GYS1^Nestin‐KO^ mice also demonstrates similar loss of microvascular MCT‐1 coverage. Images represent a 3D reconstruction of 40‐μm z‐stack. Scale bars, 10 μm. (B) MCT‐1 microvascular coverage in fold change over vehicle (saline) treated mice and WT controls both hemispheres were quantified semi‐stereologically (*n* = 3 animals per group, described in Methods, Two‐way analysis of Kruskal‐Wallis and Mann–Whitney *U*, *n* = 3; *p < 0.05). Circle: *N* = 1, female (♀), Square: *N* = 2, male (♂), Triangle: *N* = 3, female (♀). Data was shown as violin‐plot/superplots with individual values. (C) Schematic representation of how peri‐microvascular glycogen regulates microvascular constrictions. In a healthy brain, glucose in astrocyte end‐feet is incorporated in glycogen granules. Under physiological conditions, glucose‐1‐phosphate is utilized from glycogen, turned into lactate and released to the peri‐microvascular milieu. Pericytes relax and increase capillary blood flow (Right panel). Diseased states such as ischemic stroke, modeled by glycogen phosphorylase inhibitor DAB administration and GYS‐1 enzyme deficiency (GYS1^Nestin‐KO^ and GYS1^Gfap‐KO^), disrupt astrocyte glycogen synthesis, utilization and lactate bioavailability to pericytes. Pericytes constrict and cerebral blow flow diminishes due to disrupted glycogen metabolism which makes brain more susceptible to ischemia. Intravenous L‐lactate and D‐Lactate, intracerebroventricular L‐lactate administration reverses pericyte‐associated capillary constriction during glycogen phosphorylase inhibition.

Since lactate administration bypassed DAB‐induced CD13‐positive pericyte‐associated microvascular constrictions, we addressed whether lactate transporters play a role in this effect. MCT‐1 (SLC16A1) is ubiquitously expressed throughout endothelial cells, whereas MCT‐12 (SLC16A2) is expressed in pericytes (Lee et al. 2022). Immunofluorescence studies demonstrated that endothelial MCT‐1 microvascular coverage was reduced in DAB‐injected mice (Figure 8A,B; DAB‐1 h: 0.606 ± 0.057, DAB‐6 h: 0.502 ± 0.0134, DAB‐24 h: 0.504 ± 0.026, Saline‐1 h: 1.018 ± 0.077‐fold/change, Kruskal‐Wallis test, *H* = 15.60, *p* = 0.0081). GYS1^Nestin‐KO^ also showed reduced MCT‐1 coverage compared to WT controls (Figure 8A,B; GYS1^Nestin‐KO^: 0.656 ± 0.018, WT: 1.008 ± 0.058, Mann–Whitney *U* test, *U* = 0, *p* = 0.025).

## 4 Discussion

The mechanisms involved in microvascular constrictions, which adversely affect tissue perfusion, have not yet been fully elucidated. We found that disruption of glycogen utilization, either pharmacologically or genetically, via DAB, GYS1^Nestin‐KO^, and GYS1^Gfap‐KO^, respectively, led to microvascular constrictions. This suggests that disturbed glycogen metabolism can cause ischemic‐like phenotype in the brain under non‐ischemic circumstances. Although it has been shown in various studies that glycogen has essential roles in the brain, investigating the regional differences has been an issue because of the difficulties of quantification, and demonstrating this in the tissue (Oe et al. 2016; Wu, Butler, and Swanson 2019; Wu, Wong, and Swanson 2019). Our ex vivo data, which was analyzed by semi‐stereological methods to overcome this regional heterogeneity, showed that CD13‐positive pericytes were localized at constriction sites in accordance with our previous results (Alarcon‐Martinez et al. 2019; Yemisci et al. 2009). In this study, mural cells were identified using a panel of established markers (CD13, PDGFR‐β, NG2). Our analysis, based on the consistent and robust signal from CD13, revealed that constrictions occurred at pericyte sites. Notably, we also observed an intriguing heterogeneity: while CD13 and NG2 labeled all constriction sites, a subset was PDGFR‐β‐ negative (Figure S2C). This pattern of differential marker expression raises important considerations regarding the diversity of mural cells involved in metabolic constrictions. The presence of PDGFR‐β‐ negative constrictions suggests that not all mural cells may respond uniformly to disruptions in peri‐microvascular glycogen metabolism. This is consistent with the known heterogeneity of mural cells, where canonical markers like PDGFR‐β are expressed at varying levels across different subtypes (Grant et al. 2019). For instance, transitional ensheathing pericytes are often characterized by high α‐SMA but can exhibit lower PDGFR‐β expression. Therefore, our findings using the robust pan‐mural marker CD13 may encompass constrictive responses from both classical capillary pericytes and other mural cell subtypes. This potential mural cell subtype heterogeneity could influence the spatial dynamics or threshold of the constrictive response. Consistent with this interpretation, reduced PDGFR‐β expression in GYS1^Nestin‐KO^ mice may indicate a possible selective vulnerability of PDGFR‐β–expressing pericytes rather than a global loss of mural cells. However, as pericyte number and subtype composition were not directly assessed, alternative explanations such as changes in marker expression levels or cellular heterogeneity cannot be excluded. The persistence of CD13‐positive pericyte‐associated constrictions suggests that mural cell populations continue to actively regulate capillary tone. Future studies employing subtype‐specific genetic models will be crucial to dissect whether susceptibility to glycogen‐derived metabolic stress is a universal feature of mural cells or is preferentially vested in specific subtypes. This heterogeneity in marker expression suggests that some microvascular constrictions may involve distinct mural cell subtypes, including transitional or ensheathing pericytes characterized by lower or absent PDGFR‐β expression (Grant et al. 2019). Importantly, regardless of the specific mural cell subtype involved, disruption of peri‐microvascular glycogen metabolism is sufficient to trigger a pathological constrictive response at mural cell sites. This finding highlights a shared metabolic vulnerability within the neurovascular unit, while leaving open the possibility that different mural cell subtypes may exhibit distinct sensitivity thresholds. Thus, mural cell subtype heterogeneity affects the interpretation of cellular identity but does not alter the central conclusion that disruption of peri‐microvascular glycogen metabolism is sufficient to induce pathological capillary constriction.

After brain ischemia, the extracellular potassium increases, which is preceded by prompt Ca^2+^ flux and a decrease in ATP, eventually inducing the activation of phosphorylase kinase (Subbarao et al. 1995). Therefore, brain glycogen is degraded to meet this sudden energy demand when the rate of glycogenolysis may rise approximately 200‐fold above that in the resting state (Dienel and Cruz 2006; Dringen et al. 1993; Pfeiffer‐Guglielmi et al. 2005). This might explain why inhibiting glycogenolysis or abolishing glycogenesis mimics brain ischemia. Interestingly, it was also demonstrated that DAB induced CD13‐positive pericyte‐associated constrictions in the brain in a reversible and time‐dependent manner.

Glycogen utilization was inhibited with DAB, and excess glycogen was detected both with PAS and non‐commercial, glycogen‐targeted specific antibodies (ESG1A9 and IV58B6) immunofluorescently around the close vicinity of CD13‐positive pericyte‐associated microvascular constrictions (Bulmer 1959; Oe et al. 2016). Preventing the utilization of energy metabolites from glycogen with DAB, there was an increase in glycogen‐related signals despite an increased number of CD13‐positive pericyte‐associated constrictions. This suggests that DAB caused increased glycogen content at the astrocyte end‐feet and around the CD13‐positive pericyte‐associated microvascular constrictions by inhibiting glycogen phosphorylase. This might be an explanation of how glycogen increases in the peri‐infarct area in accordance with a recent study linking the relationship between increased glycogen in the peri‐infarct area of human, monkey, and mouse brain and glycogen phosphorylase activation via an insulin‐mediated mechanism in the cerebral ischemia–reperfusion model (Cai et al. 2020).

We also found that there was a decrease in the amount of glycogen around microvessels, especially where constrictions took place similar to those reported for ischemic retinal microvessels (Alarcon‐Martinez et al. 2019). As a result of 2 h of ischemia, glycogen around the microvascular niche was rapidly depleted in the infarct core, causing CD13‐positive mural cell–associated constrictions, consistent with the involvement of pericyte‐lineage cells, in line with previous findings showing reduced glycogen in PAS‐stained ischemic tissue (Hackett et al. 2016). The constriction of microvessels induced by ischemia was shown to be correlated with the reduction of perivascular glycogen in retinal glycogen, showing that in early phases of ischemic stroke, glycogen utilization plays an important role in the functional regulation of the neurogliovascular unit in microcirculatory disorders (Kilic et al. 2018; Yemisci et al. 2009). Of note, the amount of glycogen was not quantified biochemically in order not to speculate since microwave fixation needs to be performed to preserve glycogen while harvesting the tissues (Oe et al. 2016; Perezleon et al. 2013; Pfeiffer‐Guglielmi et al. 2005; Swanson 1992).

In addition, DAB‐injected and GYS1^Nestin‐KO^ mice displayed different mean regional cortical blood flow (rCBF) alteration maps compared to controls after inducing permanent middle cerebral artery occlusion (MCAo). Regional cerebral blood flow (rCBF) decreased significantly in the ischemic core compared to controls in both pharmacological and genetic models. Interestingly, the percentage of reduction of overall rCBF in the core areas of both DAB‐treated and GYS1^Nestin‐KO^ mice was much lower than in controls. We might speculate that GYS1^Nestin‐KO^ mice displayed much lower basal capillary blood flow under normal circumstances because DAB treatment caused significant rCBF reduction, and MCAo induced less rCBF reduction at core (medial) areas in vehicle‐treated controls. These findings warrant further cerebral blood flow studies. On the other hand, peri‐infarct areas did not show a significant decrease in average blood supply as in the controls. This was further supported by an increased ischemic infarct volume in these mice even after 2 h of ischemia.

Moreover, the peri‐microvascular glycogen utilization in the brain has been shown to significantly aggravate ischemia‐induced persistent CD13‐positive pericyte‐associated microvascular constrictions.

Importantly, interpretations related to astrocyte‐specific metabolic support and astrocyte–pericyte coupling are primarily based on findings from the GYS1^Gfap‐KO^ model, which lacks baseline microvascular structural alterations and therefore provides a more appropriate framework to assess astrocyte‐derived metabolic mechanisms. In contrast, sustained glycogen phosphorylase inhibition in the broader genetic and pharmacological models resulted in constrictions throughout the MCA territory, thereby diminishing ischemic tolerance and leading to increased infarct volume. On the contrary, one previous study showed that inhibiting glycogen phosphorylase with CP‐316819 attenuated ischemic tissue damage and reduced the infarct size (Suh et al. 2007). These seemingly contradictory results may be explained by differences in the pharmacological properties and ischemia‐dependent efficacy of CP‐316819. In other words, CP‐316819 treatment first increased the glycogen content in the brain, and this met the energy requirements longer than controls by allowing the cells to utilize glycogen when ischemia was induced (Obel et al. 2012).

Based on reports suggesting that lactate derived from glycogen stored in astrocytic processes may sustain glutamatergic neuronal synapses—a concept referred to as the “Astrocyte–Neuron Lactate Shuttle (ANLS)” (Kong et al. 2017; Suzuki et al. 2011)—we hypothesized that astrocytic end‐feet surrounding the microvascular wall might also support pericytes in maintaining capillary patency, particularly under ischemic conditions where energy demands are not met (Figures 5E–F and 6). Consistent with this, astrocytes with intact plasma membranes, and thus viable and resistant to hypoxia in the ischemic area, were positive for glycogen (Gurer et al. 2009), and we found that L‐lactate reversed DAB‐induced CD13‐positive pericyte‐associated microvascular constrictions, suggesting that pericyte contractility is influenced by the availability of releasable lactate derived from astrocytic glycogen stores (Figure 6). It should be noted, however, that the ANLS remains a debated hypothesis, as several studies have questioned whether astrocyte‐to‐neuron lactate shuttling is a predominant mechanism of brain energy metabolism, proposing instead that neurons themselves can directly perform glycolysis and contribute to lactate production (Hertz 2004; Tang 2018). Thus, while our findings are consistent with an ANLS‐like mechanism in the context of pericyte regulation, they should be interpreted within the framework of this ongoing debate.

To enhance clinical relevance, we also tested intravenous (i.v.) administration of L‐lactate alongside intracerebroventricular (i.c.v.) delivery. Both L‐lactate and D‐lactate reversed DAB‐induced constrictions via i.v. injection, although only L‐lactate acted as a microvascular vasodilator when delivered i.c.v. (Figure 7). This highlights the importance of the administration route in determining therapeutic outcomes. Previous work (Castillo et al. 2015) demonstrated dual roles for L‐ and D‐lactate in ischemia, with D‐lactate showing partial agonist activity at the G protein‐coupled receptor 81 (GPR81) receptor. Our findings suggest that D‐lactate dehydrogenase activity in brain endothelial cells may contribute to these effects. Moreover, our in vivo experiments following glycogen phosphorylation support earlier studies linking lactate metabolism to microvascular contractility.

Exogenous L‐lactate administration reversing microvascular constriction in ischemic stroke highlights the complex role of pericyte metabolism in vascular regulation. The contrasting outcomes between ischemic stroke and tumors likely arise from differences in pericyte metabolic states and vascular dysfunction progression. In tumors, HK2‐driven glycolysis enhances pericyte contractility in a chronically hypoxic environment, allowing reversible vascular abnormalities. In ischemic stroke, ATP depletion and mitochondrial dysfunction from oxygen and glucose deprivation lead to irreversible pericyte constriction, exacerbating tissue damage. L‐lactate appears to rescue pericytes by restoring ATP levels, reducing excessive contractility, and reversing microvascular constrictions. Beyond its role as a metabolic substrate, lactate may also function as a signaling molecule through the G protein‐coupled receptor 81 (GPR81/HCAR1), which has been increasingly implicated in cerebral ischemia (Colucci et al. 2023). Activation of GPR81 by lactate has been shown to induce pericyte constriction in the kidney during ischemia, suggesting a potential conserved, receptor‐mediated mechanism for lactate in vascular regulation across different organs (Jones et al. 2020). While our study did not directly investigate GPR81 signaling, the reversal of constrictions by exogenous L‐lactate could potentially involve both metabolic support and modulation of such receptor‐mediated pathways. It is important to note that Jones et al. (2020) reported sexual dimorphism in the renal pericyte response to GPR81 activation. Although our study used mice of both sexes, we did not observe overt differences in the lactate‐induced reversal of constrictions; nevertheless, a potential sex‐dependent effect in the brain warrants specific investigation in future studies. The most prominent change we observed in relation to lactate handling was in the expression of the lactate transporter MCT‐1 (Figure 8; Figure S6), highlighting the primary importance of lactate shuttling in our model. Therefore, the beneficial effects of lactate in our system are likely dominated by its metabolic role and transport via MCT‐1, without excluding a potential complementary signaling role via GPR81 that may become more critical under different pathological contexts or exhibit sexual dimorphism. These findings are consistent with the notion that ischemic pericytes, which undergo metabolic collapse, may be more responsive to lactate than tumor pericytes, which sustain glycolysis‐driven activity. To avoid overinterpretation, we note that the present study does not distinguish between endothelial‐ and astrocyte‐derived lactate signaling to pericytes. Comprehensive analysis of cell‐type‐specific lactate transport mechanisms, including astrocytic MCT‐4, represents an important direction for future work.

The key difference is the metabolic flexibility of pericytes: tumor pericytes maintain glycolysis‐driven contractility, while ischemic pericytes progress from reversible to irreversible dysfunction. L‐lactate's ability to reverse ischemia‐induced constrictions demonstrates the potential for metabolic interventions to address microvascular dysfunction in stroke. We also performed hexokinase‐2 (HK2) immunostaining to reveal whether DAB administration and genetic perturbation of GYS‐1 induced alterations in HK2 expression in Figure S6. Preliminary results showed increased HK2 expression in CD13‐positive pericytes in both pharmacological and genetic models, as in Figure S6. Although revealing the underlying mechanisms requires further research, we can speculate that glycogen stores in astrocytes sustain pericyte contractibility, thus ensuring adequate tissue perfusion, especially under demanding conditions. These results were in accordance with the previous study demonstrating that the application of L‐lactate reversed the decrease in the cortical spreading depolarisation threshold induced by DAB (Kilic et al. 2018) and reduced infarct size with improved neurological outcomes after MCAo (Berthet et al. 2009; Castillo et al. 2015; Lee et al. 2022). To avoid overinterpretation, we note that the present study does not directly quantify intracerebral lactate levels following peripheral administration, nor does it assess stereoisomer‐specific lactate kinetics or toxicity. The differential effects of L‐ and D‐lactate observed here therefore provide functional rather than biochemical evidence, and direct measurements of brain lactate dynamics will be important for future studies.

Our findings on mural cell–associated constrictions in response to metabolic stress, including those involving pericyte‐lineage cells, contribute to an evolving understanding of mural cell function. The role of pericytes in regulating cerebral blood flow has been a subject of active debate. While some studies, under specific physiological conditions, have questioned their contractile role (Hill et al. 2015), a substantial and consistent body of evidence demonstrates that pericytes are central to microvascular dysfunction in pathology. Foundational work established that neurotransmitters evoke pericyte‐mediated capillary constriction in situ and that pericytes contribute to the vascular response to ischemia (Peppiatt et al. 2006). This was directly extended by studies showing that ischemia evokes capillary constriction by pericytes, leading to long‐lasting flow deficits (Hall et al. 2014; Yemisci et al. 2009), a mechanism conserved in other organs like the heart and kidney (Freitas and Attwell 2022; O'Farrell et al. 2017). Our data, showing that disruption of peri‐microvascular glycogen metabolism—a novel metabolic insult—induces pathological constriction, integrate seamlessly into this established paradigm of pathological pericyte contractility. They identify a new upstream metabolic pathway (glycogen‐lactate axis) by which energy failure can engage this critical mechanism, distinct from debates about their role in physiological neurovascular coupling. This underscores pericytes as key metabolic sensors and effectors in the neurovascular unit, whose dysfunction underlies microcirculatory failure.

We addressed that peri‐microvascular glycogen plays an important role in the components that can cause a susceptibility for many CNS diseases, primarily in the very early ischemic phase by mediating CD13‐positive pericyte‐associated microvascular constrictions, thus adequate tissue perfusion (Figure 6). Our study on the pericyte behavior in relation to glycogen metabolism and cerebral blood flow in health and disease may also give additional insight to the scientists who have been researching glycogen and its importance in the pathophysiology of many diseases such as ischemic stroke, migraine, cognitive dysfunction, and memory loss (Kilic et al. 2018; Oe et al. 2016; Pellerin and Magistretti 1994; Pellerin et al. 1997). These observations, specifically focusing on microvascular niches, also warrant further studies to understand the metabolic crosstalk between glial end‐feet and pericytes in the control of microvascular function.

A methodological consideration of this study is that pericyte identification relied on a convergent multi‐marker and morphological approach—based on independent single‐marker stainings—rather than dual‐labeling strategies that provide single‐cell resolution. As acknowledged in the field, commonly used pericyte markers such as CD13, PDGFR‐β, and NG2 can label contiguous mural cell populations, limiting definitive assignment at the level of individual cells (Grant et al. 2019). While this established approach is robust for identifying mural cell localization and capillary constriction sites within the neurovascular unit, future studies employing dual‐reporter mouse models or sparse labeling strategies will be valuable to resolve single‐pericyte dynamics in vivo. Furthermore, the observed heterogeneity in PDGFR‐β labeling at constriction sites suggests that the responses described here may encompass different mural cell subtypes, such as ensheathing pericytes, highlighting potential subtype‐specific vulnerabilities to metabolic stress.

In addition, regional cerebral blood flow (rCBF) measurements were not performed in the astrocyte‐specific GYS1^Gfap‐KO^ model. Although microvascular constriction analyses provide a direct and sensitive readout of pericyte‐associated capillary regulation, future studies combining microvascular assessments with rCBF measurements in this model will be important to further define how astrocytic glycogen depletion translates into regional cerebral perfusion changes. Notably, the primary aim of the present study was to identify microvascular and mural cell–level mechanisms underlying metabolic stress–induced constrictions, for which capillary‐level analyses provide a more direct and sensitive readout than global perfusion measurements.

## Author Contributions

Gökhan Uruk performed the experiments, contributed to data analysis, design of the study, and drafting the manuscript, and prepared the figures. Buket Donmez‐Demir contributed to in vivo Laser Speckle Contrast imaging, ICT image calculations, generating data and revising the manuscript. Sinem Yilmaz‐Ozcan supervised the experiments and contributed to data analysis and the design of the study. Canan Cakir‐Aktas contributed to in vivo MCA occlusion experiments. Aslıhan Taskiran‐Sag contributed to in vivo experiments, drafting the manuscript, and figures. Gokce Gurler contributed to histochemical labeling, analysis of microvascular constrictions, and drafting the manuscript and figures. Otto Baba and Tsuyoshi Morita provided glycogen‐specific antibody IV58B6. Jordi Duran and Joan J. Guinovart established the GYS‐1^Nestin‐KO^ and GYS1^Gfap‐KO^ transgenic line, provided the mouse colony, and contributed to the design of the study. Hulya Karatas and Turgay Dalkara contributed to the hypothesis and design of the study. Muge Yemisci conceived the hypothesis, contributed to the design of the study, supervised the experiments and data analysis, and wrote the manuscript. All authors discussed the results and commented on the manuscript.

## Funding

This work was supported by Hacettepe University Scientific Research Projects Coordination Unit (THD‐2017‐14025, TDK‐2019‐17652).

## Conflicts of Interest

The authors declare no conflicts of interest.

## Supporting information


**Video S1:** Glycogen‐specific antibody IV58B6 reveals peri‐microvascular glycogen. Double Immunofluorescence labeling with anti‐glycogen antibody IV58B6 (green), and CD13 (magenta) in naive mice. Anti‐glycogen antibody reveals substantial deposition around CD13+ pericytes and microvessels in the merged 3D video. The perinuclear cellular anti‐glycogen antibody‐related signal intensity reveals the astrocytes and some perivascular mural cells. The video represents a 3D reconstruction of 40‐μm z‐stack.

**Figure S1:** This figure explains the temporal relationship of regional cerebral blood flow (rCBF) changes in each experimental paradigm in Figure 1. Representative time‐lapse rCBF changes in ischemic core (black line) and peri‐infarct (red line) areas of intracerebroventricular (i.c.v) (A) Saline‐injected (B) DAB‐injected ischemic mice via middle cerebral artery occlusion (MCAo). When i.c.v. injections and MCAo inductions are performed shown as arrows. Black box delineates the ischemic period of speckle contrast imaging in right panels. (B) Representative image taken during MCAo. Red dots delineate the area of ischemic core after successful clotting of the middle cerebral artery (MCA). Representative time‐lapse rCBF changes in ischemic core (black line) and peri‐infarct (red line) areas of (C) wild‐type (WT) and GYS1^Nestin‐KO^ mice via middle cerebral artery occlusion (MCAo). When MCAo inductions are performed shown as arrows. *n* = 3 for each group.
**Figure S2:** Quantification of Microvascular Constrictions. (A) Illustrative images of mouse brains: sagittal view (left panel), coronal view (right panel). Modified from George Paxinos' mouse brain atlas. Ten vertical black lines represent each coronal section taken for semi‐stereological quantification (left panel). Vertical and horizontal black lines indicate each area at 40× magnification under a fluorescent microscope (360 × 240 μm). Blue rectangles demonstrate the 10 areas in which quantification takes part (ROI size: 240 × 160 μm). (B) *Lycopersicon esculentum* Lectin (Lectin) labeled microvascular constrictions shown as arrows. Parameters defining constrictions are explained with further details in Methods. Images represent a 3D reconstruction of a 40‐μm z‐stack. Scale bars, 10 μm. (C) Double labeling with Lectin (upper panels‐green) and CD13 (left lower panel‐red), platelet growth factor receptor‐beta (PDGFR‐β) (middle lower panel‐red), and neural glial antigen‐2 (NG2) (right lower panel‐red) demonstrates pericyte‐mediated microvascular constrictions (arrows). Images represent a 3D reconstruction of a 40‐μm z‐stack. Scale bars, 10 μm. (D) Lectin‐labeled microvascular constrictions in flash‐frozen (with Liquid nitrogen) brain sections are shown as arrows. Scale bars, 10 μm. (E) Western blotting of wild‐type (WT), GYS1^Gfap‐KO^, and GYS1^Nestin‐KO^ mice shows successful downregulation of glycogen synthase‐1 (GYS1) in both transgenics, which results in substantially reduced platelet‐derived growth factor receptor‐beta (PDGFR‐β) expression in GYS1^Nestin‐KO^ mice. (F) Immunofluorescence study demonstrating GYS1 expression in wild‐type (WT), GYS1^Gfap‐KO^, and GYS1^Nestin‐KO^ mice. Arrows show peri‐microvascular GYS1 expression in WT but not the transgenic mice. Scale bars, 10 μm. (G) Periodic acid Schiff (PAS) staining in GYS1^Nestin‐KO^ mice showing no peri‐microvascular glycogen stores. Scale bars, 10 μm. (H) Glycogen‐specific IV58B6 (green) and Lectin (red) double labeling reveals no peri‐microvascular glycogen stores in GYS1^Nestin‐KO^ mice. Scale bars, 10 μm.
**Figure S3:** Quantification of Microvascular Branching. (A*) Lycopersicon esculentum* Lectin (Lectin) labeled microvascular tree in wild‐type (WT), GYS1^Gfap‐KO^, and GYS1^Nestin‐KO^ mice. Only GYS1^Nestin‐KO^ mice demonstrate a substantial loss of microvascular coverage, which indicates a developmental defect. *n* = 3 animals per group. Mann–Whitney *U*, *n* = 3; *p < 0.05. Data was shown as violin‐plot/superplots with individual values. Red circle: *n* = 1, female (♀), Green square: *n* = 2, male (♂), Yellow triangle: *n* = 3, female (♀).
**Figure S4:** Immunofluorescence with anti‐glycogen antibodies IV58B6 and ESG1A9 in wild‐type (WT) mice. (A) Double labeling with IV58B6 (left panel‐green) and *Lycopersicon esculentum* Lectin (Lectin) (middle panel‐red). Anti‐glycogen antibody reveals substantial deposition around microvessels and GFAP+ astrocytic cytoplasms in merged image (right panel). (B) Double labeling with ESG1A9 (left panel‐green) and Lectin (middle panel‐red). Anti‐glycogen antibody reveals substantial deposition around microvessels in merged image (right panel). White boxes and smaller inserts show astrocyte end‐feet surrounding microvessel segments. Images represent 3D reconstruction of 40‐μm z‐stack. Scale bars, 10 μm.
**Figure S5:** Immunofluorescence with anti‐glycogen antibody ESG1A9 in Saline, DAB‐injected, naïve and ischemic mice. (A) Illustrative image of coronal section of mouse brain including the middle cerebral artery (MCA) territory of intracerebroventricular (i.c.v) DAB‐injected mice. The red box represents the area where the images were taken. (B) Double labeling with ESG1A9 (left panel‐green) and Lectin (middle panel‐red) in naïve mice. Anti‐glycogen antibody reveals substantial deposition around microvessels in merged image (right panel) as shown with arrows. (C) Illustrative image of coronal section of mouse brain including MCA territory in naïve and ischemic mice. (D) Double labeling with ESG1A9 (green‐left panel) and Lectin (red‐middle panel) around core area in ischemic mice 2 h after permanent middle cerebral artery occlusion (MCAo). The loss of anti‐glycogen antibody‐related signal intensity reveals the coincident nature of microvessel constrictions (arrows) and glycogen depletion (right panel). Images represent a 3D reconstruction of 40‐μm z‐stack. Scale bars, 10 μm.
**Figure S6:** Double immunofluorescence labeling of CD13‐positive pericytes and (A) G protein‐coupled receptor 81 (GPR81), (B) Monocarboxylate transporter‐12 (MCT12), and (C) Hexokinase‐2 (HK2) in intracerebroventricular (i.c.v) saline, DAB (sacrificed after 1 h, 6 h, and 24 h (shown left to right, respectively)) injected Swiss albino, wild‐type (WT) and GYS1^Nestin‐KO^ mice. Arrows show GPR81, MCT12 or HK2‐expressing pericytes. Images represent a 3D reconstruction of 40‐μm z‐stack. Scale bars, 10 μm. *n* = 3 animals per group.
**Figure S7:** The whole Western blotting against (A) NG2, (B) CD13, (C) PDGFR‐β, (D) GYS‐1 and (E) Beta‐actin of wild‐type (WT), GYS1^Gfap‐KO^, and GYS1^Nestin‐KO^ mice, which is shown in Figure S2E.

## Data Availability

All aggregated data supporting the findings of this study are included in the main text, figures, or supporting information. Primary data (including images and spreadsheets) are available from the corresponding author upon reasonable written request. This study does not report any original code. Additional information related to this work is available from the corresponding author upon request.
