# Supplementary material for: Peri‐Microvascular Glycogen and Lactate Regulate Capillary Constrictions and Ischemia Outcome in Mice
Source: J Neurochem. 2026 Apr 16;170(4):e70430. doi: 10.1111/jnc.70430 (PMC13085847; doi:10.1111/jnc.70430)
Supplement: Supplementary file 2 — Figure S1: This figure explains the temporal relationship of regional cerebral blood flow (rCBF) changes in each experimental paradigm in Figure 1. Representative time‐lapse rCBF changes in ischemic core (black line) and peri‐infarct (red line) areas of intracerebroventricular (i.c.v) (A) Saline‐injected (B) DAB‐injected ischemic mice via middle cerebral artery occlusion (MCAo). When i.c.v. injections and MCAo inductions are performed shown as arrows. Black box delineates the ischemic period of speckle contrast imaging in right panels. (B) Representative image taken during MCAo. Red dots delineate the area of ischemic core after successful clotting of the middle cerebral artery (MCA). Representative time‐lapse rCBF changes in ischemic core (black line) and peri‐infarct (red line) areas of (C) wild‐type (WT) and GYS1Nestin‐KO mice via middle cerebral artery occlusion (MCAo). When MCAo inductions are performed shown as arrows. n = 3 for each group. Figure S2: Quantification of Microvascular Constrictions. (A) Illustrative images of mouse brains: sagittal view (left panel), coronal view (right panel). Modified from George Paxinos' mouse brain atlas. Ten vertical black lines represent each coronal section taken for semi‐stereological quantification (left panel). Vertical and horizontal black lines indicate each area at 40× magnification under a fluorescent microscope (360 × 240 μm). Blue rectangles demonstrate the 10 areas in which quantification takes part (ROI size: 240 × 160 μm). (B) Lycopersicon esculentum Lectin (Lectin) labeled microvascular constrictions shown as arrows. Parameters defining constrictions are explained with further details in Methods. Images represent a 3D reconstruction of a 40‐μm z‐stack. Scale bars, 10 μm. (C) Double labeling with Lectin (upper panels‐green) and CD13 (left lower panel‐red), platelet growth factor receptor‐beta (PDGFR‐β) (middle lower panel‐red), and neural glial antigen‐2 (NG2) (right lower panel‐red) demonstrates pericyte‐medi [file JNC-170-e70430-s002.docx]

**SUPPLEMENTARY FIG. 1**

**
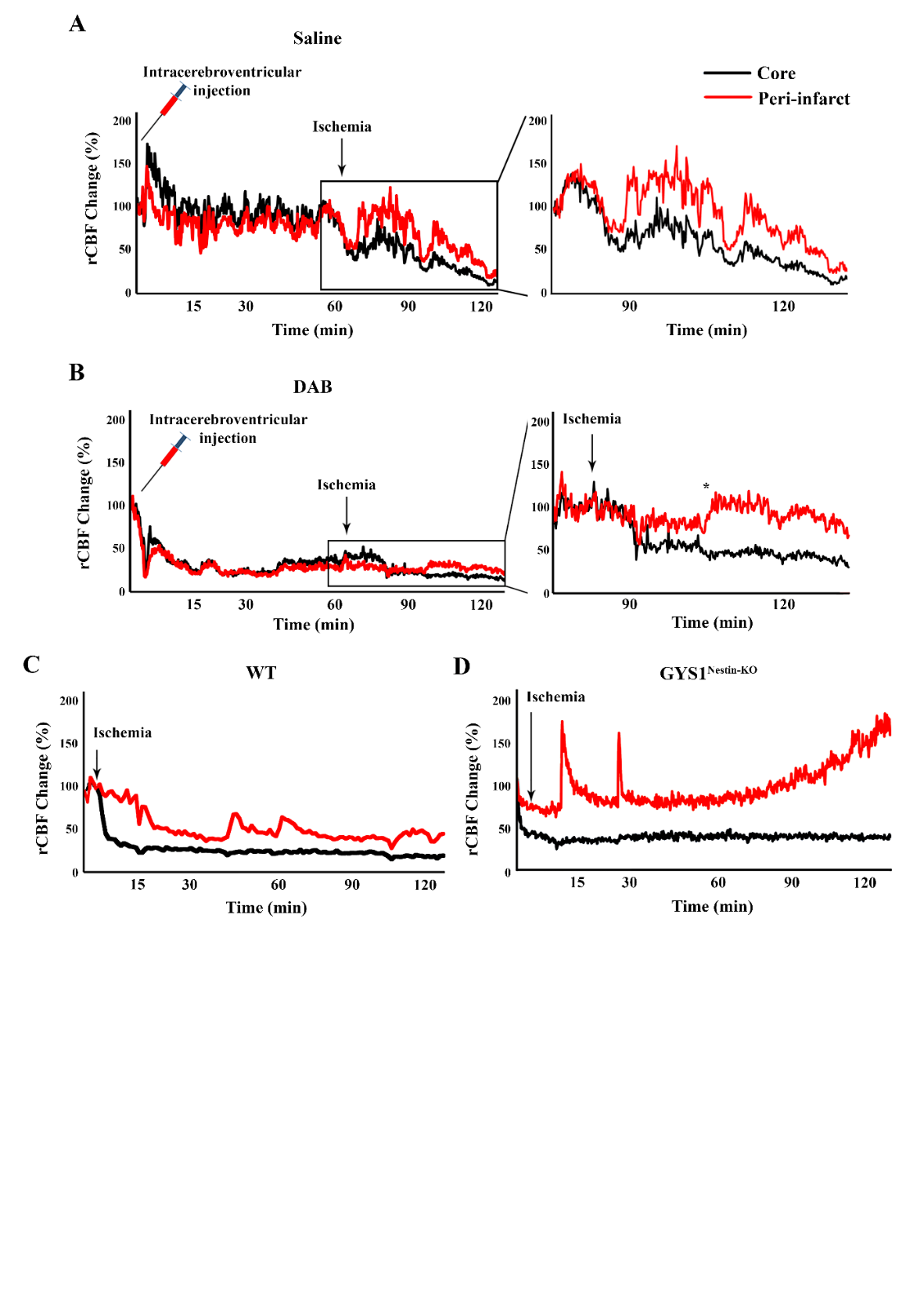
**

This figure explains the temporal relationship of regional cerebral blood flow (rCBF) changes in each experimental paradigm in Fig. 1. Representative time-lapse rCBF changes in ischemic core (black line) and peri-infarct (red line) areas of intracerebroventricular (i.c.v) (A) Saline-injected (B) DAB-injected ischemic mice via middle cerebral artery occlusion (MCAo). When i.c.v injections and MCAo inductions are performed shown as arrows. Black box delineates the ischemic period of speckle contrast imaging in right panels. (B) Representative image taken during MCAo. Red dots delineate the area of ischemic core after successful clotting of the middle cerebral artery (MCA). Representative time-lapse rCBF changes in ischemic core (black line) and peri-infarct (red line) areas of (C) wild-type (WT) and GYS1^Nestin-KO^ mice via middle cerebral artery occlusion (MCAo). When MCAo inductions are performed shown as arrows. n = 3 for each group.

**SUPPLEMENTARY FIG. 2**

**
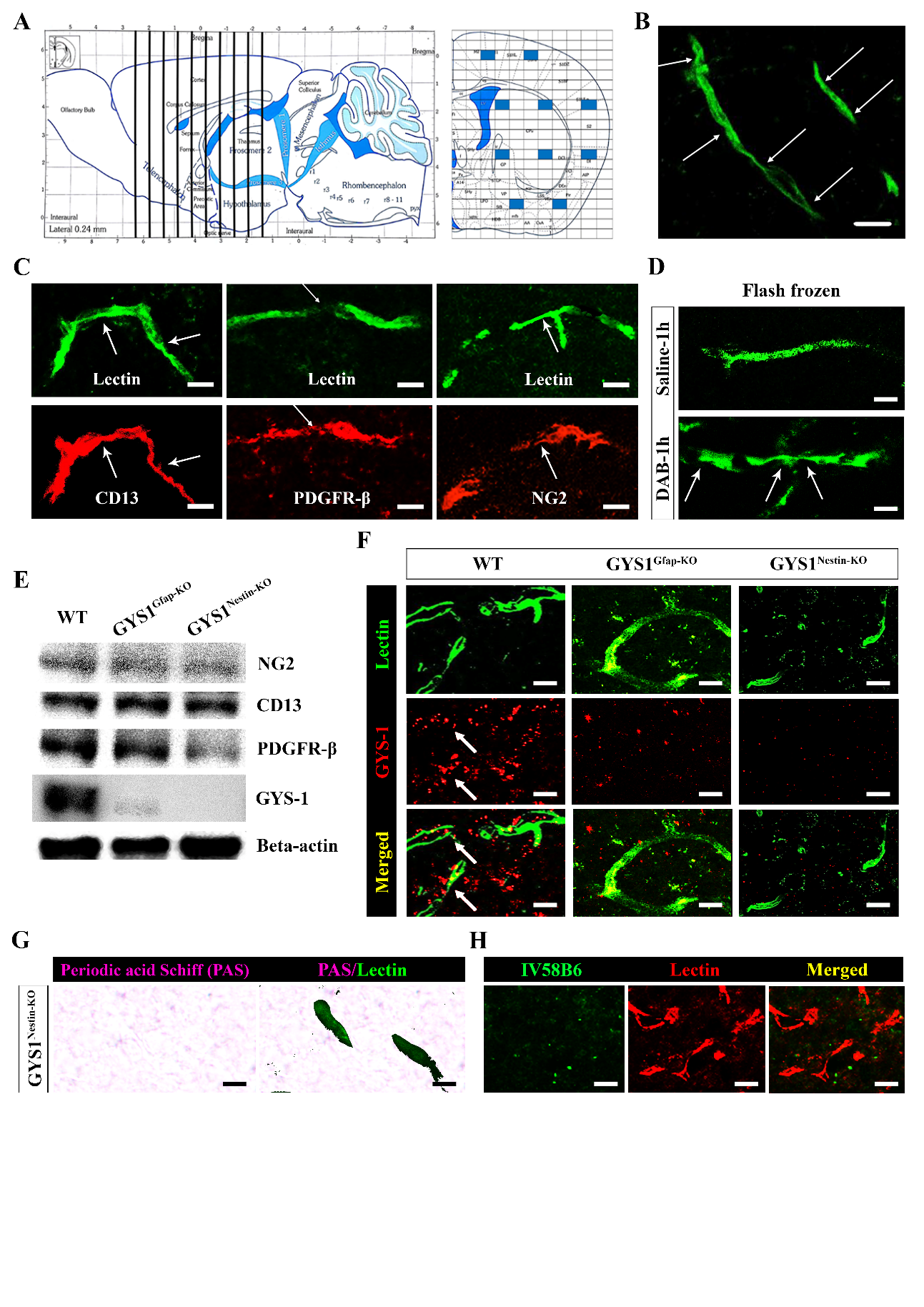
**

Quantification of Microvascular Constrictions. (A) Illustrative images of mouse brains: sagittal view (left panel), coronal view (right panel). Modified from George Paxinos' mouse brain atlas. Ten vertical black lines represent each coronal section taken for semi-stereological quantification (left panel). Vertical and horizontal black lines indicate each area at 40x magnification under a fluorescent microscope (360 μm × 240 μm). Blue rectangles demonstrate the 10 areas in which quantification takes part (ROI size: 240 μm × 160 μm). (B) *Lycopersicon esculentum* Lectin (Lectin) labelled microvascular constrictions shown as arrows. Parameters defining constrictions are explained with further details in Methods. Images represent a 3D reconstruction of a 40-μm z-stack. Scale bars, 10 μm. (C) Double labelling with Lectin (upper panels-green) and CD13 (left lower panel-red), platelet growth factor receptor-beta (PDGFR-β) (middle lower panel-red), and neural glial antigen-2 (NG2) (right lower panel-red) demonstrates pericyte-mediated microvascular constrictions (arrows). Images represent a 3D reconstruction of a 40-μm z-stack. Scale bars, 10 μm. (D) Lectin-labelled microvascular constrictions in flash-frozen (with Liquid nitrogen) brain sections are shown as arrows. Scale bars, 10 μm. (E) Western blotting of wild-type (WT), GYS1^Gfap-KO^, and GYS1^Nestin-KO^ mice shows successful downregulation of glycogen synthase-1 (GYS1) in both transgenics, which results in substantially reduced platelet-derived growth factor receptor-beta (PDGFR-β) expression in GYS1^Nestin-KO^ mice. (F) Immunofluorescence study demonstrating GYS1 expression in wild-type (WT), GYS1^Gfap-KO^, and GYS1^Nestin-KO^ mice. Arrows show peri-microvascular GYS1 expression in WT but not the transgenic mice. Scale bars, 10 μm. (G) Periodic acid Schiff (PAS) staining in GYS1^Nestin-KO^ mice showing no peri-microvascular glycogen stores. Scale bars, 10 μm. (H) Glycogen-specific IV58B6 (green) and Lectin (red) double labelling reveals no peri-microvascular glycogen stores in GYS1^Nestin-KO^ mice. Scale bars, 10 μm.

**SUPPLEMENTARY FIG. 3**

**
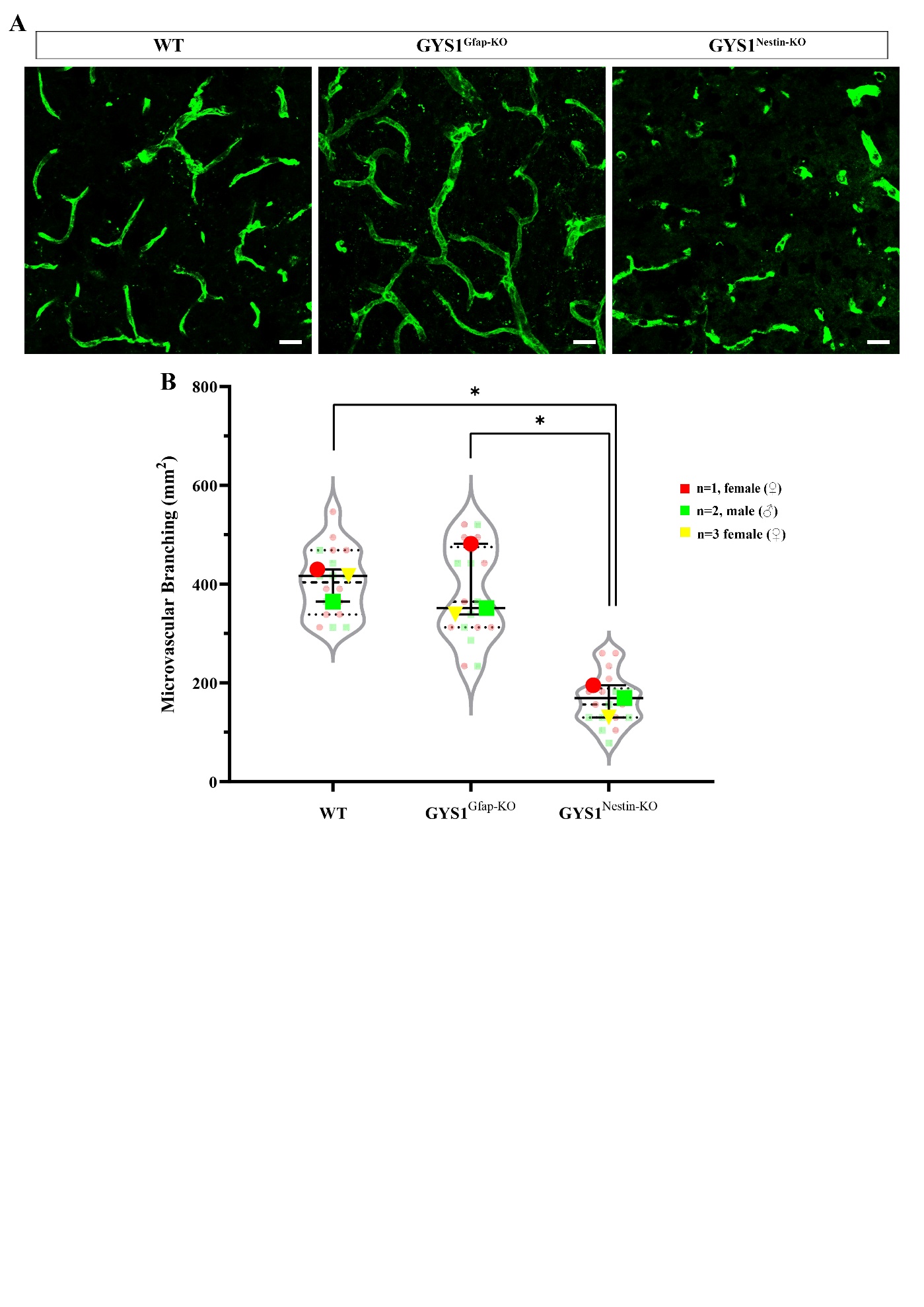
**

Quantification of Microvascular Branching. (A*) Lycopersicon esculentum* Lectin (Lectin) labelled microvascular tree in wild-type (WT), GYS1^Gfap-KO^, and GYS1^Nestin-KO^ mice. Only GYS1^Nestin-KO^ mice demonstrate a substantial loss of microvascular coverage, which indicates a developmental defect. n = 3 animals per group. Mann-Whitney U, n = 3; *P < 0.05. Data was shown as violin-plot/ superplots with individual values. Red circle: n=1, female (♀), Green square: n=2, male (♂), Yellow triangle: n=3, female (♀).

**SUPPLEMENTARY FIG. 4**

**
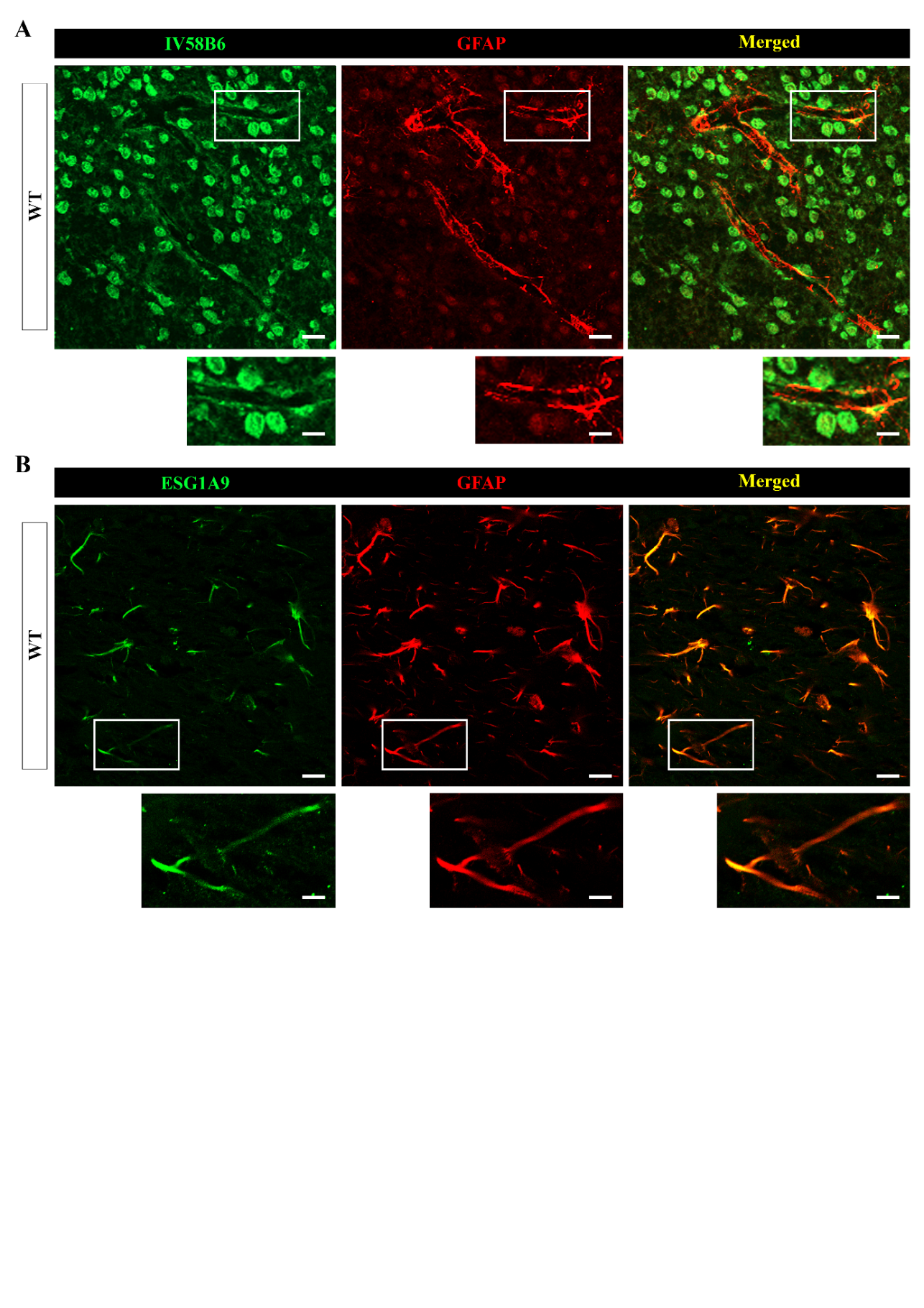
**

Immunofluorescence with anti-glycogen antibodies IV58B6 and ESG1A9 in wild-type (WT) mice. (A) Double labelling with IV58B6 (left panel-green) and *Lycopersicon esculentum* Lectin (Lectin) (middle panel-red). Anti-glycogen antibody reveals substantial deposition around microvessels and GFAP+ astrocytic cytoplasms in merged image (right panel). (B) Double labelling with ESG1A9 (left panel-green) and Lectin (middle panel-red). Anti-glycogen antibody reveals substantial deposition around microvessels in merged image (right panel). White boxes and smaller inserts show astrocyte end-feet surrounding microvessel segments. Images represent 3D reconstruction of 40-μm z-stack. Scale bars, 10 μm. **SUPPLEMENTARY FIG. 5**

**
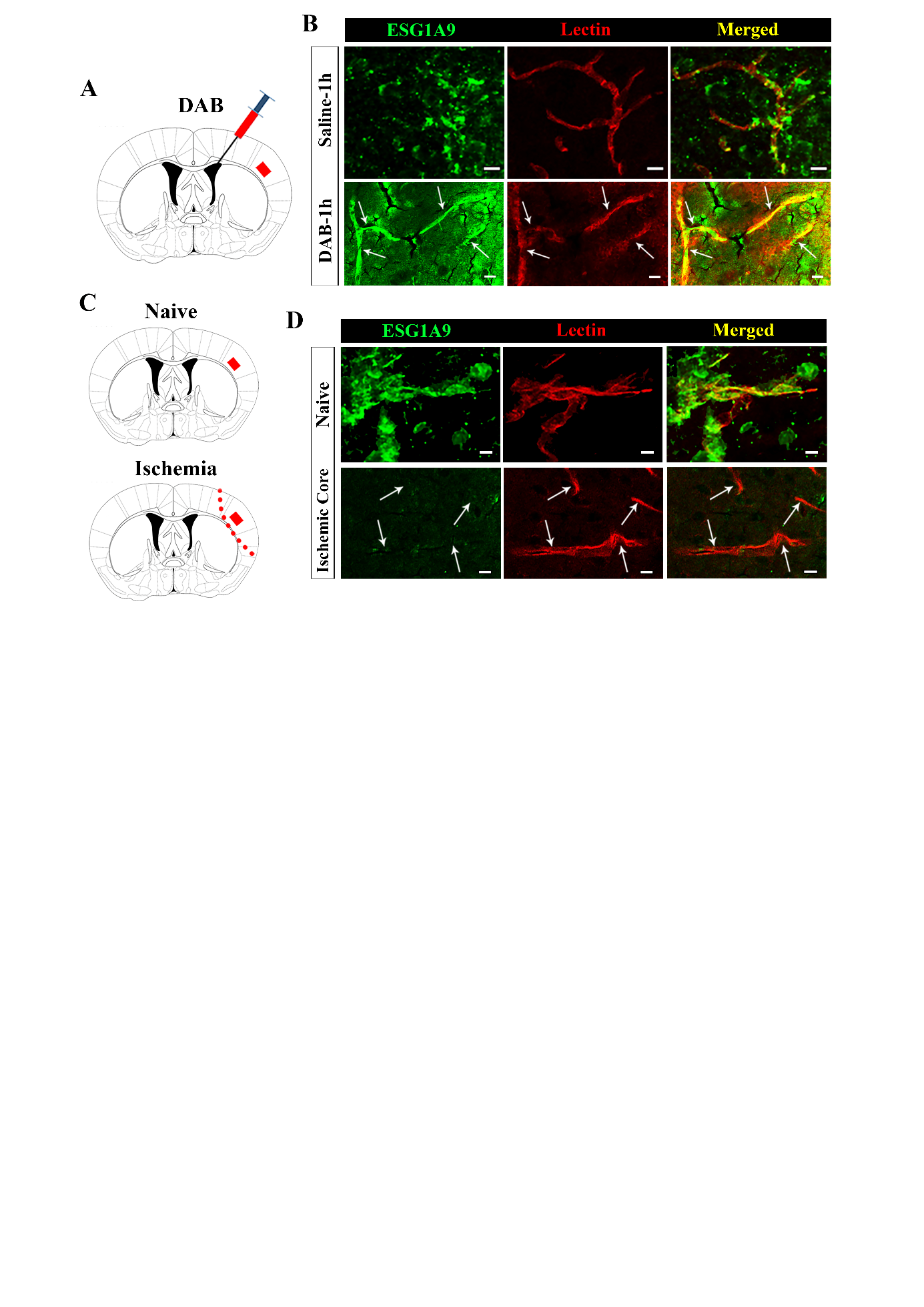
**

Immunofluorescence with anti-glycogen antibody ESG1A9 in Saline, DAB-injected, naïve and ischemic mice. (A) Illustrative image of coronal section of mouse brain including the middle cerebral artery (MCA) territory of intracerebroventricular (i.c.v) DAB-injected mice. The red box represents the area where the images were taken. (B) Double labelling with ESG1A9 (left panel-green) and Lectin (middle panel-red) in naïve mice. Anti-glycogen antibody reveals substantial deposition around microvessels in merged image (right panel) as shown with arrows. (C) Illustrative image of coronal section of mouse brain including MCA territory in naïve and ischemic mice. (D) Double labelling with ESG1A9 (green-left panel) and Lectin (red-middle panel) around core area in ischemic mice 2 hours after permanent middle cerebral artery occlusion (MCAo). The loss of anti-glycogen antibody-related signal intensity reveals the coincident nature of microvessel constrictions (arrows) and glycogen depletion (right panel). Images represent a 3D reconstruction of 40-μm z-stack. Scale bars, 10 μm.

**SUPPLEMENTARY FIG. 6**


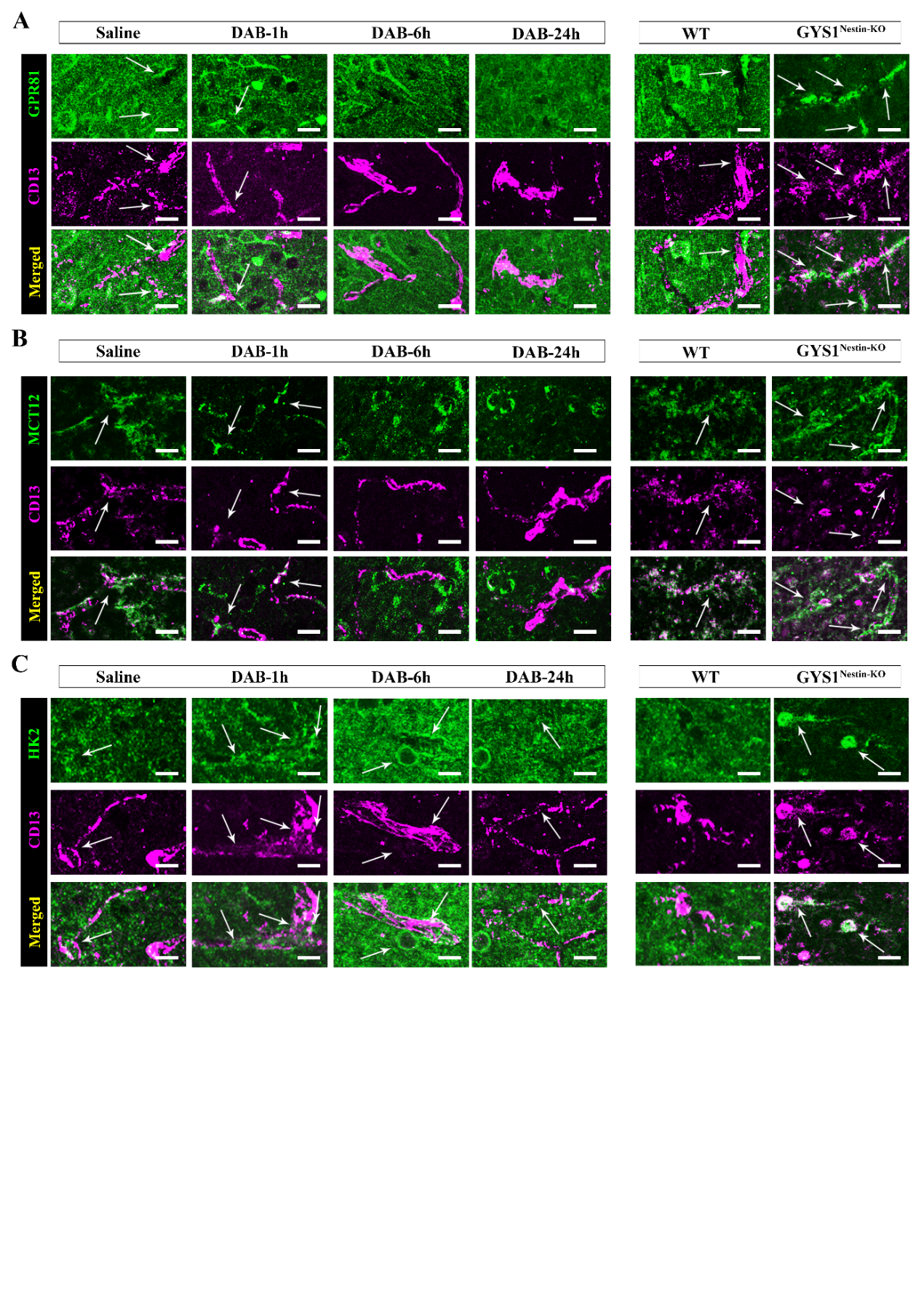


Double immunofluorescence labelling of CD13-positive pericytes and (A) G protein-coupled receptor 81 (GPR81), (B) Monocarboxylate transporter-12 (MCT12), and (C) Hexokinase-2 (HK2) in intracerebroventricular (i.c.v) saline, DAB (sacrificed after 1h, 6h, and 24h (shown left to right, respectively)) injected Swiss albino, wild-type (WT) and GYS1^Nestin-KO^ mice. Arrows show GPR81, MCT12 or HK2-expressing pericytes. Images represent a 3D reconstruction of 40-μm z-stack. Scale bars, 10 μm. n = 3 animals per group.

**SUPPLEMENTARY FIG. 7**


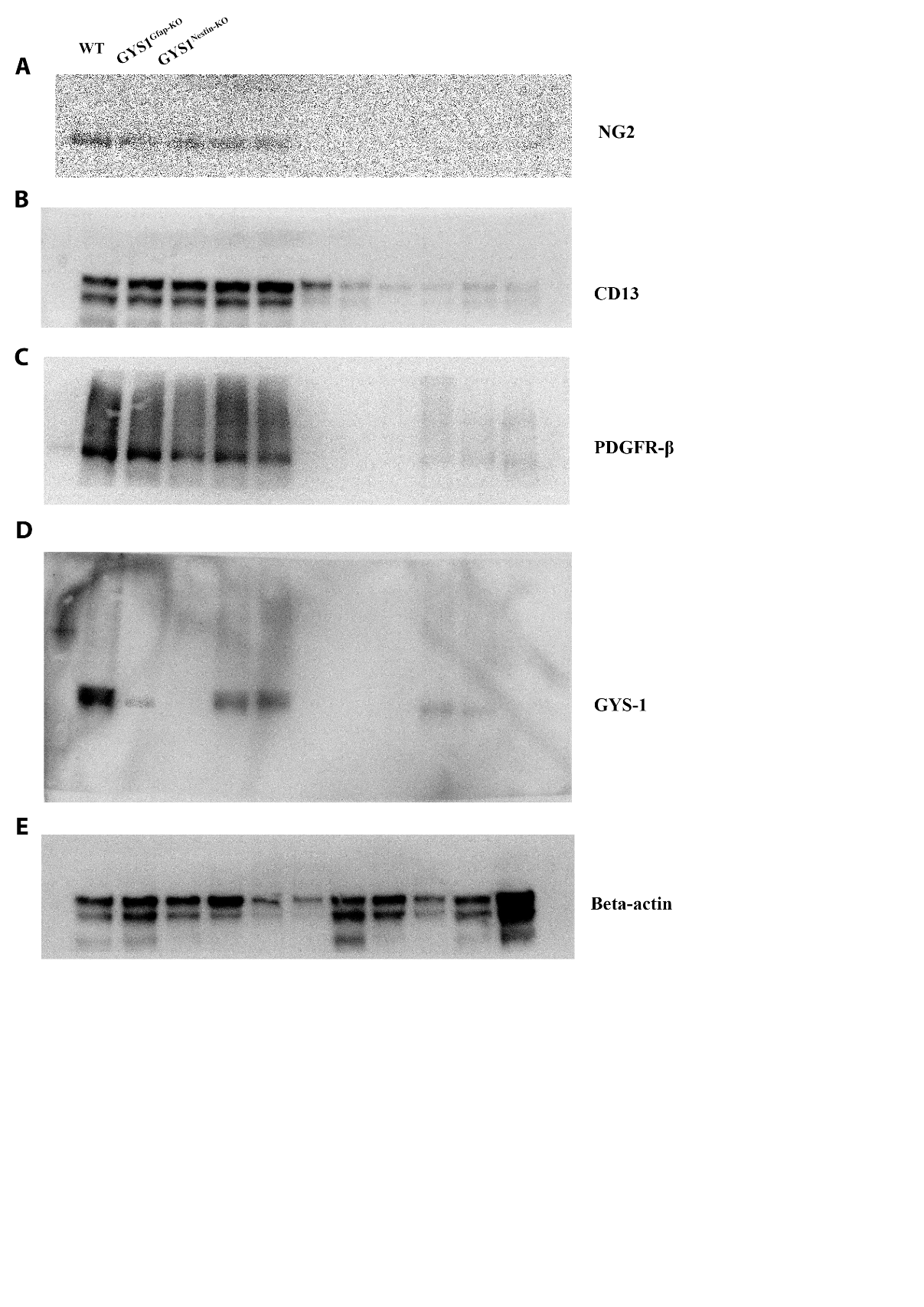


The whole Western blotting against (A) NG2, (B) CD13, (C) PDGFR-β, (D) GYS-1 and (E) Beta-actin of wild-type (WT), GYS1^Gfap-KO^, and GYS1^Nestin-KO^ mice, which is shown in Supplementary Figure 2E.
